# Hypergravity reduces F-actin accumulation in osteoclasts, with attenuated bone resorption

**DOI:** 10.1371/journal.pone.0351542

**Published:** 2026-06-16

**Authors:** Natsuhiro Takahashi, Akihiko Fujita, Yuki Azetsu, Akiko Karakawa, Mie Myers, Masamichi Takami, Masahiro Chatani

**Affiliations:** 1 Department of Pharmacology, Graduate School of Dentistry, Showa Medical University, ‌‌1-5-8 Hatanodai, Shinagawa-ku, Tokyo, Japan; 2 Pharmacological Research Center, Showa Medical University, 1-5-8 Hatanodai, ‌‌Shinagawa-ku, Tokyo, Japan; 3 Department of Medical and Dental Cooperative Dentistry, Graduate School of Dentistry, Showa Medical University, 2-1-1 Kitasenzoku, Ota-ku, Tokyo, Japan; 4 Department of Orthodontics, Graduate School of Dentistry, Showa Medical University, 2-1-1 Kitasenzoku, Ota-ku, Tokyo, Japan; CHU Caen: Centre Hospitalier Universitaire de Caen, FRANCE

## Abstract

Bone loss occurs in astronauts during prolonged spaceflight, thus indicating the sensitivity of skeletal homeostasis to altered gravitational environments. Previous studies have shown that microgravity affects osteoclast differentiation and bone resorption, which suggests that osteoclasts possess mechanisms to sense and respond to gravity-generated mechanical forces. For testing of the related mechanisms, hypergravity can be experimentally reproduced with use of a centrifuge. In the present study, osteoclasts derived from mouse bone marrow were subjected to hypergravity under three conditions: 30G exposure using a non-CO_2_ centrifuge system, and short- or long-term exposure to 3G or 5G using an incubator-compatible centrifuge system. Cytoskeletal organization and resorptive function were assessed using TRAP (tartrate-resistant acid phosphatase) staining, F-actin visualization, and dentin pit assays. In addition, phosphoproteomic analysis was performed after short-term exposure to 5G hypergravity. Hypergravity exposure for as brief as 30 minutes compromised F-actin ring integrity, reduced fluorescence intensity, and promoted nuclear repositioning toward actin rings, whereas tubulin and vinculin localization remained unchanged, and the structural alterations corresponded to attenuated resorption pit formation. Quantitative phosphoproteomic profiling revealed coordinated hypergravity-dependent changes in phosphorylation across multiple cellular modules, including cytoskeletal organization, membrane trafficking, intracellular signaling, and nuclear regulatory pathways. Together, these results indicate that osteoclasts are sensitive to gravity-generated mechanical loading, with hypergravity rapidly modifying F-actin-associated cytoskeleton properties and reprogramming phosphorylation-dependent signaling networks, ultimately attenuating bone-resorptive activity. These findings provide mechanistic insight into how osteoclasts respond to altered gravitational loading conditions and have implications for skeletal adaptation during spaceflight and under altered mechanical loading conditions on Earth.

## Introduction

The microgravity environment of spaceflight is associated with marked bone loss in astronauts during long-term missions [1,2], which has primarily been attributed to increased bone resorption and decreased bone formation [3–6]. Skeletal adaptation to altered gravity is therefore considered a consequence of changes in mechanical loading at both tissue and cellular levels. In contrast, astronauts are also exposed to transient hypergravity (3-5G, i.e., 3- to 5-fold of Earth gravity) during rocket launch and re-entry, and thereby experience both significantly elevated and reduced gravitational forces. In addition to spaceflight, humans encounter hypergravity in aircraft maneuvering, centrifuge-based training, and acceleration environments, where short-term gravitational overload represents a physiologically relevant mechanical stimulus [7]. Under these conditions, gravity acts as an acceleration that generates mechanical forces within tissues and cells. Consequently, cells respond not to gravity directly, but to gravity-induced forces such as compression, tension, shear stress, and hydrostatic pressure, depending on their spatial orientation and physical constraints [8]. These conditions therefore provide a valuable framework for investigating how gravity-generated mechanical forces regulate cellular behavior.

Osteoclasts are multinucleated giant cells responsible for bone resorption. In response to macrophage colony-stimulating factor (M-CSF) and receptor activator of nuclear factor-κB ligand (RANKL), these cells differentiate from bone marrow-derived mononuclear precursors [9] and are involved in bone resorption via sealing zone (actin ring) formation and pit generation on dentin or bone substrates [10,11]. In this process, cell adhesion signaling plays an essential role in maintaining RANK expression and enabling RANKL-induced osteoclast differentiation, as reported previously [12]. Furthermore, osteoclasts are highly sensitive to mechanical stimuli [13,14] and their responses to changes in forces generated by gravity are receiving increased attention. Several studies have investigated the effects of microgravity on osteoclast differentiation and function, using diverse experimental models that have yielded partially divergent observations. Smith summarized evidence indicating that microgravity alters osteoclast cytoskeletal organization and resorptive capacity, while emphasizing variability depending on experimental models and exposure duration [15]. Spaceflight experiments demonstrated that microgravity directly enhances osteoclast differentiation and bone resorption in vitro, accompanied by increased pit formation [16]. Together, these studies suggest that altered gravitational environments modulate osteoclastogenesis and resorptive activity through both direct cellular responses and indirect systemic influences; however, the precise mechanisms by which osteoclasts sense gravity-generated mechanical forces remain incompletely understood.

Sealing zone (actin rings) in osteoclasts are known to be essential structures that provide adhesion to bone surfaces as well as resorptive activity [17], and function as central elements of intracellular mechanotransduction. The osteoclast cytoskeleton consists of structural and signaling proteins, including tubulin, vinculin, c-Src, and integrins [18–20], which provide regulation of adhesion, signaling, and cellular morphology. Dynamic modulation of F-actin organization, rather than gross structural disruption, is closely associated with changes in osteoclast function [17]. Furthermore, osteoclast function is modulated by osteoblast-osteoclast communication, which influences resorption activity and spatial organization of actin structures, highlighting the importance of cell-cell interactions in skeletal homeostasis [21,22]. Accumulating evidence indicates that unloading-induced muscle atrophy plays a pivotal role in skeletal deterioration under microgravity through bone-muscle crosstalk. While muscle unloading is a critical contributor to bone loss under microgravity [23], the direct gravity-dependent responses of bone-resorbing cells themselves remain less well characterized. In particular, how osteoclast cytoskeletal architecture and signaling pathways respond to distinct modes of gravity-generated mechanical loading has not been fully elucidated.

For the present study, a centrifugal device was used to experimentally reproduce hypergravity, then the effects on osteoclast structure and function were examined. In this context, hypergravity represents an experimental condition that increases gravity-generated mechanical forces acting on cells, rather than a simple alteration of the gravity vector itself. Hypergravity was employed not as an inverse substitute for microgravity, but as a controllable mechanical perturbation that allows precise examination of rapid cellular responses to increased gravity-generated mechanical loading. Previous physiological studies using centrifuged mice have made important contributions to understanding bone metabolism under hypergravity [24,25]. Building upon these achievements, we performed cell-level analyses to elucidate the direct effects of hypergravity on osteoclasts. For bone resorption assessment, osteoclast monocultures and osteoclast-osteoblast co-cultures were used, while cytoskeletal organization, nuclear localization, and phosphoproteomic profiles were analyzed under various hypergravity conditions (3G, 5G, 30G). In addition, inverted loading configurations (1G, −1G, and −3G) were used to evaluate the influence of force orientation relative to the substrate. Given the experimental configuration, cells cultured on substrate surfaces are expected to experience a combination of compressive and shear-related forces under hypergravity, rather than compression alone. We show that hypergravity rapidly modifies F-actin organization and nuclear positioning, and is associated with attenuation of bone-resorptive activity, depending on the magnitude and duration of gravitational loading.

Additionally, we performed phosphoproteomic analysis of alterations in proteins related to cytoskeletal regulation and mechanotransduction, because phosphorylation is also known to play crucial roles in osteoclast activation [26]. By integrating functional and molecular analyses, this study aims to clarify how osteoclasts directly sense and respond to altered gravitational loading conditions. Specifically, we focus on the relationship between gravity-generated mechanical forces and intracellular signaling pathways governing cytoskeletal dynamics and resorptive function. Together, the findings indicate that hypergravity inhibits osteoclast bone-resorptive function by modulating actin assembly and protein phosphorylation, thus providing mechanistic insights relevant to bone remodeling under altered gravity conditions.

## Materials and methods

### Animal experiments

All animal experiments were performed in accordance with protocols approved by the Animal Care and Use Committee of Showa Medical University (approval numbers: 224056, 224066). C57BL/6J male mice were purchased from Japan SLC (Shizuoka, Japan) and maintained under a specific pathogen-free (SPF) condition at the animal facility of Showa Medical University. They were housed under a 12-hour light/dark cycle with controlled temperature and humidity, and provided access to food and water ad libitum. For tissue collection, mice were euthanized by cervical dislocation performed by trained personnel, in accordance with institutional guidelines. Because all subsequent experiments were conducted in vitro using isolated cells, no anesthesia or analgesia was administered. To alleviate animal suffering, mice were handled gently, monitored daily, and euthanized promptly when tissues were required, minimizing the duration of handling and stress.

### Isolation of osteoclast progenitor cells

Bone marrow cells were isolated from femurs and tibias of 5- to 8-week-old male C57BL/6J mice on Day 0, and cultured in α-minimum essential medium (α-MEM; Fujifilm Wako, Osaka, Japan) supplemented with 20 ng/mL M-CSF (R&D Systems, Minneapolis, USA) at 37°C in a 5% CO_2_ atmosphere. After 3 days of culture, non-adherent cells were removed, and adherent bone marrow-derived macrophages (BMMs) were used as osteoclast progenitors in the assays described below.

### Medium and reagents

The base medium was α-MEM or Leibovitz’s L-15 (Fujifilm Wako, Osaka, Japan), while supplements included 10% fetal bovine serum (FBS; Gibco, Grand Island, USA) and penicillin-streptomycin mixed solution (Nacalai Tesque, Kyoto, Japan). Differentiation factors used were M-CSF at 20 ng/mL and RANKL at 100 ng/mL (R&D Systems, Minneapolis, USA). Vitamin C (ascorbic acid; Fujifilm Wako, Osaka, Japan) at 50 mg/L was added only in pit assays performed under atmospheric CO_2_ conditions (0.04%), because Leibovitz’s L-15 medium, which is suitable for CO_2_-independent culture, does not contain Vitamin C, unlike α-MEM. Medium was replaced every 3–4 days (specific schedule indicated in each experimental section).

### Hypergravity devices and calibration

Hypergravity cultures were performed using Zeromo CL-5100 (Kitagawa, Hiroshima, Japan) and LIX-140SP (Tomy Seiko, Tokyo, Japan) devices. CL-5100 is able to generate different gravity levels depending on the culture vessel, with 209 rpm producing 3G for 24-well plates (Falcon, Corning, USA) and 5G for 25-mL flasks (Falcon, Corning, USA). The LIX-140SP generates 30G at 390 rpm. Hypergravity exposure was performed at 37°C, with CO_2_ concentrations specified for each experiment. Because the LIX-140SP does not allow CO_2_ control during centrifugation, experiments conducted using this device were performed under atmospheric CO_2_ conditions (0.04%) with CO_2_-independent Leibovitz’s L-15 medium, whereas experiments using the CL-5100 were conducted in a standard 5% CO_2_ environment. Importantly, within each experimental setup, both the 1G control and corresponding hypergravity groups were cultured under identical CO_2_ conditions, and gravity-dependent comparisons were therefore not confounded by differences in CO_2_ concentration.

### Pit assay: Bone marrow macrophage monocultures (1G vs 30G)

Osteoclast resorption activity on dentin slices was evaluated. On Day 3, BMMs were detached and switched to Leibovitz’s L-15 medium containing M-CSF (20 ng/mL), RANKL (100 ng/mL), and vitamin C (50 mg/L). Dentin slices (diameter 5 mm) were pre-fixed to the bottom of 96-well plates (Thermo Fisher Scientific, Waltham, USA) using Medical Adhesive Silicone Type A (Dow Corning, Midland, USA), then BMMs were seeded at 2.0 × 10^4^ cells/well. The cultures were maintained at 37°C under 0.04% CO_2_ at 1G or 30G until Day 16, with medium replaced every 3 days. On Day 16, cells were fixed with 4% paraformaldehyde (PFA; Muto Pure Chemicals, Tokyo, Japan).

### Pit assay: BMM and primary osteoblast co-cultures (1G vs 30G)

Primary osteoblasts were isolated from calvaria of neonatal C57BL/6J mice by treatment with 0.1% collagenase and 0.2% dispase. Isolated osteoblasts were cryopreserved in liquid nitrogen until use. 4 days before BMM seeding (designated Day −4, relative to BMM isolation), osteoblasts were thawed and cultured in α-MEM with medium changes every 3 days. Cells were expanded by passaging as needed. On Day 3 of BMMs culture, osteoblasts were detached using 0.25% Trypsin-EDTA (Fujifilm Wako, Osaka, Japan). and co-seeded together with BMMs onto dentin slices pre-fixed to the bottom of 96-well plates at a total density of 2.0 × 10^4^ cells/well. Leibovitz’s L-15 medium supplemented with 100 ng/mL of prostaglandin E_2_ (Sigma-Aldrich, St. Louis, USA), 100 ng/mL of 1α,25(OH)₂D₃ (Fujifilm Wako, Osaka, Japan), and 50 mg/L of vitamin C was used, and cultures were maintained at 37°C with 0.04% CO_2_ at 1G or 30G until Day 14, with medium replaced every 3 days. On Day 14, cells were fixed with 4% PFA.

### Pit assay: RepCell-derived osteoclasts (1G vs 3G)

BMMs were seeded onto RepCell (CellSeed, Tokyo, Japan) culture dishes on Day 3 and differentiated in α-MEM containing M-CSF (20 ng/mL) and RANKL (100 ng/mL) until Day 14. Cells were then detached at 4°C for 10 minutes and seeded onto dentin slices (diameter 5 mm) in 24-well plates at 2.0 × 10^4^ cells/well. Pit assays were performed under 1G or 3G at 37°C with 5% CO_2_ until Day 20, with medium replaced every 4 days. On Day 20, cells were fixed with 4% PFA.

### Pit assay: RepCell-derived osteoclasts under inverted gravity conditions (1G, −1G, and −3G)

BMMs were seeded onto RepCell culture dishes on Day 3 and differentiated in α-MEM containing M-CSF (20 ng/mL) and RANKL (100 ng/mL). On Day 13, mature osteoclasts were detached from the RepCell dishes and seeded onto dentin slices (diameter 5 mm) pre-fixed to the bottom of 24-well plates at 2.0 × 10⁴ cells/well (cell replating). To ensure firm attachment of osteoclasts to the dentin surface and prevent detachment during subsequent inverted culture or centrifugation, cells were incubated overnight under standard upright 1G conditions at 37°C in 5% CO₂ prior to gravity loading. On Day 14, cultures were subjected to conventional upright culture (1G), inverted static culture (−1G), or inverted centrifugation culture (−3G) for 3 days at 37°C in 5% CO₂. The medium was replaced every 3 days during the culture period. At the end of the experiment, cells were fixed with 4% PFA for subsequent staining and analysis.

### Short-term (30 minutes) hypergravity exposure (1G vs 30G, 1G vs 3G, 1G vs 5G)

BMMs were detached on Day 3 and reseeded in medium containing M-CSF (20 ng/mL) and RANKL (100 ng/mL). The medium was replaced on Day 6.

For 1G vs 30G exposure, BMMs were seeded into 48-well plates at 7.0 × 10^4^ cells/well and cultured under 0.04% CO_2_ conditions. On Day 7, cells were exposed to 30G for 30 minutes at 37°C using the LIX-140SP hypergravity device. Control groups were maintained under identical culture conditions and exposed to 1G for 30 minutes. For 1G vs 3G exposure, BMMs were seeded into 24-well plates at 2.0 × 10^4^ cells/well and cultured under 5% CO_2_ conditions. On Day 7, cells were exposed to 3G for 30 minutes at 37°C using the CL-5100 device. Control groups were maintained under identical culture conditions and exposed to 1G for 30 minutes. Cells were fixed with 4% PFA immediately after exposure. For exploratory experiments (S4 Fig., S5 Fig.), BMMs were seeded into 25-mL flasks at 2.0 × 10^6^ cells/flask on Day 3. Due to the larger medium volume and accelerated differentiation, hypergravity exposure was performed on Day 6. Cells were exposed to 5G for 30 minutes at 37°C under 5% CO_2_. In some experiments, cells exposed to 5G for 30 minutes were subsequently returned to 1G conditions for an additional 30 minutes before fixation.

### Rationale for seeding density and plate format

Seeding densities and plate formats were selected based on the requirements of each experimental setup and device configuration. Importantly, the aim of this study was not to compare absolute osteoclast differentiation efficiency across different plate formats or cell densities, but to evaluate the relative effects of different gravity conditions under identical culture conditions. Therefore, all gravity-dependent comparisons were performed strictly within the same plate format and seeding density (e.g., 1G vs. 30G, 1G vs. 3G, or 1G vs. 5G), and no direct comparisons were made across different well sizes or culture formats. Similarly, fixation time points were determined individually for each experimental system based on osteoclast maturation and assay requirements, such as differences in differentiation kinetics and the time required to achieve stable osteoclast morphology and resorption activity in each culture system. Gravity-dependent comparisons were always performed within the same system and time point, and no direct comparisons were made across different fixation time points.

### Short-term (30 minutes) inverted gravity exposure (1G, −1G, and −3G)

BMMs were detached on Day 3 and reseeded into 24-well plates at 2.0 × 10^4^ cells/well in α-MEM containing M-CSF (20 ng/mL) and RANKL (100 ng/mL). The medium was replaced on Day 6. On Day 7, osteoclasts were subjected to conventional upright culture (1G), inverted static culture (−1G), or inverted centrifugation culture (−3G) for 30 minutes at 37°C in 5% CO₂ using the CL-5100 device. For the −3G condition, culture plates were mounted in an inverted orientation so that centrifugal force was directed away from the culture substrate. Control cells (1G) and inverted static controls (−1G) were maintained under otherwise identical environmental conditions. Immediately after exposure, cells were fixed with 4% PFA and processed for subsequent morphological analyses, including Rhodamine-phalloidin staining and TRAP staining.

### Phosphoproteomic analysis (1G vs 5G)

BMMs were detached on Day 3 and seeded at 3.0 × 10^6^ cells/25-mL flask (first experiment) in medium containing M-CSF and RANKL. On Day 6, cells were exposed to 5G in the CL-5100 at 37°C with an atmosphere containing 5% CO_2_ for 30 minutes, while the control cells were maintained at 1G. Cells were washed with PBS (-) (Shimadzu Diagnostics, Tokyo, Japan) and inactivated with 0.1 vol% trifluoroacetic acid-acetonitrile (ACN-0.1% TFA, Kanto Kagaku, Tokyo, Japan), then scraped and placed in conical tubes. Samples were then obtained and frozen in liquid nitrogen, and stored at −80°C. In a second independent phosphoproteomic experiment, BMMs were seeded at 1.59 × 10^7^ cells per 25-mL flask, and phosphoproteomic analysis was performed using three independent flasks per condition (n = 3). All experimental procedures, including culture conditions, gravity exposure, sample preparation, and fixation, were identical to those used in the first experiment. The first experiment (n = 1 per condition) was conducted as an exploratory screening to identify gravity-responsive phosphorylation events, while the second experiment (n = 3 per condition) was performed to confirm the reproducibility of phosphorylation changes observed in the screening analysis. The analyses were conducted by Kazusa Genome Technologies (Kisarazu, Japan).

### Rhodamine-phalloidin staining

F-actin structures were visualized using Rhodamine-phalloidin staining. Cells were fixed with 4% PFA, washed with PBS (-), permeabilized with 0.1% Triton X-100 (MP Biomedicals, Santa Ana, USA) for 15 minutes, and blocked with 1% bovine serum albumin (BSA; Sigma-Aldrich, St. Louis, USA) for 30 minutes. Phalloidin, Rhodamine X conjugated (Fujifilm Wako, Osaka, Japan) was applied for 2 hours at room temperature. After washing, fluorescence images were acquired with a BZ-X810 fluorescence microscope (Keyence, Osaka, Japan). All images used for qualitative and quantitative comparisons were acquired within the same experiment under identical acquisition settings. Image contrast and intensity were not arbitrarily adjusted during image acquisition or subsequent analysis.

### Antibody staining

Cells were fixed and permeabilized as detailed above, blocked with 1% BSA, and incubated with the following primary antibodies: α-Tubulin (Alexa Fluor 488, clone DM1A; Abcam, Cambridge, UK), Vinculin (Alexa Fluor 488, clone EPR8185; Abcam, Cambridge, UK), c-Src (Alexa Fluor 488, clone B-12; Santa Cruz Biotechnology, Dallas, USA), and Integrin β3 (Rabbit polyclonal; StressMarq Biosciences, Victoria, Canada), Alexa Fluor 488-conjugated antibodies were used directly, while other primary antibodies were detected using donkey anti-rabbit IgG (Alexa Fluor 488; Abcam, Cambridge, UK). Nuclei were stained with DAPI (Fujifilm Wako, Osaka, Japan). Microscopic images were acquired using a BZ-X810 fluorescence microscope. Immunofluorescence images represent independent single-color staining experiments performed with individual antibodies. Each antibody was applied and imaged separately, and these experiments were not designed for multiplex imaging or co-localization analysis.

### TRAP staining

TRAP staining was performed to assess osteoclast differentiation and activity. Cells were fixed with ethanol/acetone for 1 minute, then washed and air-dried. Fast Red Violet LB Salt (Sigma-Aldrich, St. Louis, USA) and Naphthol AS-MX phosphate (Sigma-Aldrich, St. Louis, USA) in 0.1 M acetate buffer (pH 5.0) with 1% N,N-dimethylformamide (Fujifilm Wako, Osaka, Japan) were applied at 37°C for 20 minutes. Stained cells were imaged using a BZ-X810 fluorescence microscope. For TRAP staining, quantitative evaluation was restricted to comparisons between the 1G and hypergravity groups within the same culture system (monoculture or co-culture). TRAP staining intensity was not quantitatively compared across different culture systems.

### Toluidine blue staining

Following the pit assay, dentin slices were stained with 1% Toluidine blue O (Polysciences, Warrington, USA) for 5 minutes and imaged using a VHX-6000 digital microscope (Keyence, Osaka, Japan).

### Quantification and image analysis

Image analysis was performed using ImageJ, ver. 1.54g (National Institutes of Health, Bethesda, USA). Osteoclast number, Rhodamine-phalloidin-positive area, TRAP-positive area, and resorption pit area were quantified using thresholding, particle analysis, and macros. Consistent image acquisition and analysis settings were applied within each experiment, with threshold values adjusted between experiments according to staining intensity and background levels. Analyses were performed using 8-bit images. All images used for quantitative and qualitative comparisons were acquired within the same experiment using fixed image acquisition settings, including exposure time, illumination intensity, and detector gain. No arbitrary contrast or brightness adjustments were applied during image acquisition or analysis. The mean fluorescence intensity of the actin ring was quantified from Rhodamine-phalloidin-stained images using raw, unadjusted images, without thresholding or binarization. For some images, osteoclast and nuclei numbers were quantified by manual counting.

### Statistical analysis

Statistical analyses were performed using JMP Pro, ver. 17.0.0 (Statistical Discovery, Durham, USA). Data are presented as mean ± standard deviation (SD), unless otherwise specified. Comparisons between two groups were made using a two-tailed Student’s t-test, whereas comparisons among three groups were conducted using one-way analysis of variance (ANOVA) followed by Tukey’s multiple comparison test. Statistical significance was set at p < 0.05. In the figures, significance is indicated as *p < 0.05, **p < 0.01, or ***p < 0.001, and comparisons showing no significant differences are indicated as n.s.

## Results

### Osteoclast bone resorption attenuated under severe hypergravity at 30G

**Fig 1:**
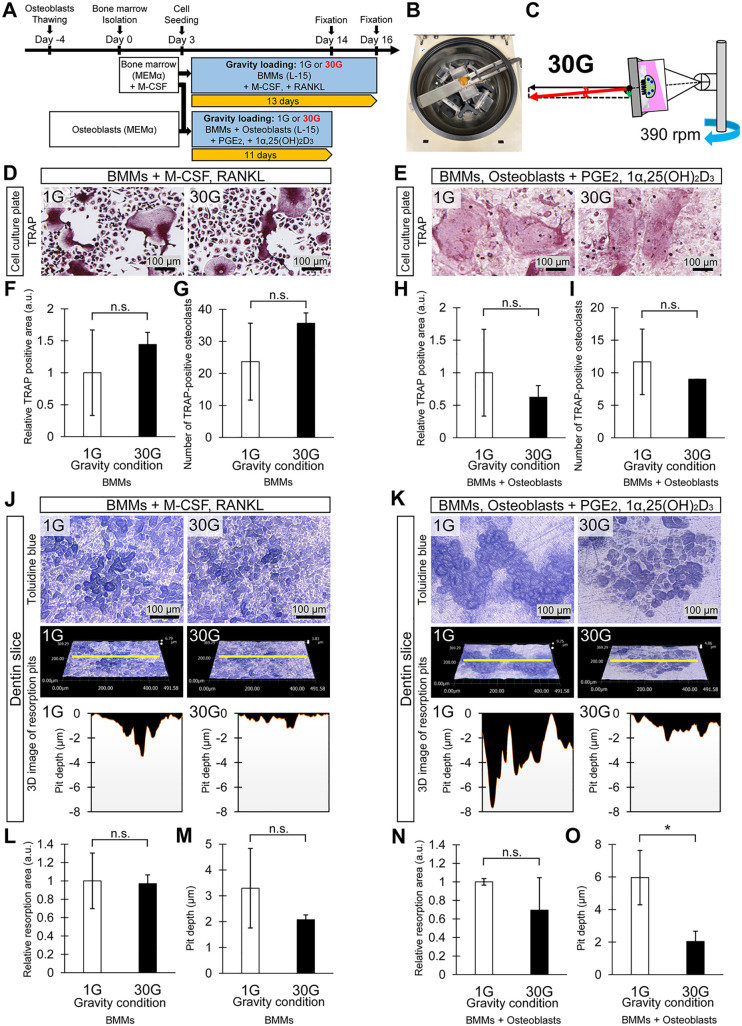
Severe hypergravity (30G) attenuates osteoclastic bone resorption on dentin. **(A)** Experimental timeline for monoculture and coculture pit assays on dentin slices. **(B)** LIX-140SP hypergravity device with mounted plate. **(C)** Schematic diagram of gravity-generated mechanical loading under hypergravity applied to osteoclasts. **(D, E)** Representative osteoclasts on culture plates stained with TRAP under monoculture **(D)** and coculture **(E)** conditions. **(F)** Quantification of TRAP-positive area in osteoclast monocultures. **(G)** Number of TRAP-positive osteoclasts larger than 100 µm in monoculture. **(H)** Quantification of TRAP-positive area in coculture. **(I)** Number of TRAP-positive osteoclasts larger than 100 µm in coculture. **(J, K)** Representative resorption pits stained with Toluidine Blue and corresponding 3D reconstructions. Yellow lines indicate the positions used for cross-sectional analysis. **(L)** Relative pit area in monoculture. **(M)** Pit depth (µm) in monoculture. **(N)** Relative pit area in coculture. **(O)** Pit depth (µm) in coculture. Relative values are normalized to the 1G control group. For F-I, ImageJ threshold values were set to 50-170, and TRAP-positive osteoclasts with a diameter ≥100 µm were analyzed. For L and N, threshold values were set to 70-140, and resorption pits with a diameter ≥10 µm and an aspect ratio <1.5 were analyzed. Scale bar: 100 µm. Error bars indicate standard deviation (SD). Statistical comparisons were performed using a two-tailed unpaired Student’s t-test. p < 0.05. Data were obtained from three independent experiments. Each data point represents one well or one dentin slice (1G and 30G, n = 3 wells or dentin slices per condition), except for the 1G coculture condition (n = 2 dentin slices). For each well or dentin slice, quantitative analyses were performed using three randomly acquired images, and the averaged value per well or dentin slice was used for statistical analysis.

To evaluate the effects of severe hypergravity on osteoclast function, BMMs were cultured either alone or in cocultures with osteoblasts under a 1G or 30G condition. TRAP staining of cell culture plates was performed to assess osteoclast differentiation, while pit assays of dentin slices were conducted to evaluate bone resorption (Fig 1A). For osteoclast monoculture experiments, bone marrow cells were isolated on Day 0 and cultured with M-CSF until Day 3, followed by culture with M-CSF and RANKL on cell culture plates or dentin slices until fixation on Day 16. For osteoclast-osteoblast coculture experiments, osteoblasts were prepared four days in advance and maintained with medium replacement until Day 3. BMMs were then added, and cocultures were maintained in the presence of prostaglandin E2 and 1α,25(OH)_2_D_3_ until fixation on Day 14. The LIX-140SP hypergravity device was set to 390 rpm and provided continuous 30G (Fig 1B, C). Under the hypergravity condition, osteoclasts in both monocultures and cocultures displayed no morphological or TRAP activity changes, indicating that hypergravity has no effect on osteoclast differentiation (Fig 1D-I). In addition, the number of TRAP-positive osteoclasts (≥100 µm) was quantified in monocultures and cocultures (Fig 1G, I). In monocultures, all osteoclasts contained three or more nuclei, confirming successful fusion. In cocultures, counting nuclei for fusion index was difficult due to overlapping cells; therefore, the number of TRAP-positive osteoclasts ≥100 µm was used instead. In pit assays, osteoclasts monocultured under 30G exhibited no significant change in pit area or pit depth (Fig 1J, L, M). In cocultures, resorption area showed no significant change, whereas pit depth was significantly reduced under 30G (Fig 1K, N, O). These findings indicate that severe hypergravity of 30G attenuates bone resorption, particularly when osteoclasts interact with osteoblasts, and that this effect is specific to pit depth rather than resorption area.

### Pronounced alterations in actin ring structure and nuclear repositioning induced by short-term 30G hypergravity

**Fig 2:**
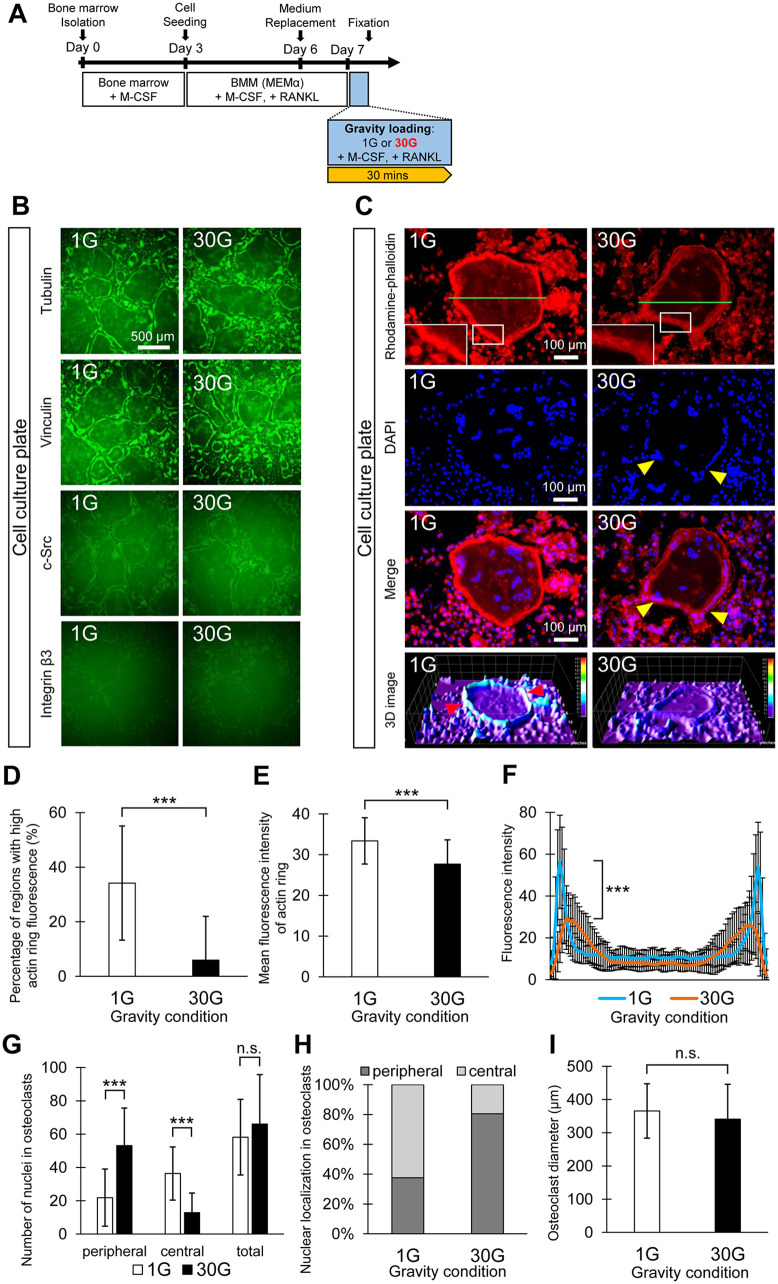
Short-term (30 minutes) severe hypergravity (30G) alters F-actin organization and nuclei positioning in osteoclasts. **(A)** Experimental timeline. Osteoclasts were exposed to 1G or 30G for 30 minutes. **(B)** Immunostaining for Tubulin, Vinculin, c-Src, and Integrin β3. Each marker was stained and imaged separately. (scale bar: 500 µm). **(C)** Rhodamine-phalloidin and DAPI staining, with merged images and 3D intensity maps. Yellow arrows indicate nuclei localized within actin rings, and red arrows indicate regions of high F-actin intensity. Scale bar: 100 µm. **(D)** Percentage of regions with high actin ring fluorescence. **(E)** Mean fluorescence intensity of actin rings. **(F)** Line-scan analysis of Rhodamine-phalloidin fluorescence intensity across actin ring cross-sections. **(G, H)** Nuclear positioning shown by representative images **(G)** and quantification of peripheral versus central nuclear localization **(H)**. **(I)** Osteoclast diameter. Error bars indicate standard deviation (SD). Statistical comparisons were performed using a two-tailed unpaired Student’s t-test. ***p < 0.001. Data were obtained from two independent experiments. Panel E was analyzed using nine wells, with three osteoclasts imaged per well (n = 27 cells). Panels D and F-I were analyzed using 16 wells, with three osteoclasts imaged per well (n = 48 cells); however, in panel F, the numbers of analyzed cells differed between conditions (1G: n = 48 cells; 30G: n = 47 cells). In all analyses, individual osteoclasts were treated as data points (n = cell number).

To investigate rapid cytoskeletal responses, osteoclasts were exposed to severe hypergravity at 30G for 30 minutes (Fig 2A). Representative microscopic images of osteoclasts immediately before and after hypergravity loading are provided (S1 Fig.). Immediately before gravity loading, actin ring organization was comparable between the 1G and 30G groups, with no apparent structural differences. In contrast, immediately after hypergravity exposure, localized alterations in the arrowhead region of the actin ring were observed in the 30G group, whereas the actin rings in the 1G group remained unchanged. Although immunofluorescence staining for cytoskeleton- and adhesion-related proteins, including Tubulin, Vinculin, c-Src, and Integrin β3 showed no obvious differences as compared with the 1G control (Fig 2B), Rhodamine-phalloidin staining indicated reduced fluorescence intensity and increased sealing zone (actin ring) width under 30G (Fig 2C-F). The percentage of regions with high actin ring fluorescence along the osteoclast circumference was significantly reduced in the 30G group compared with the 1G control (Fig 2D). In addition, the mean fluorescence intensity of the actin ring was significantly decreased under 30G conditions (Fig 2E). Consistent with these findings, cross-sectional analysis of F-actin fluorescence intensity along the green line in Fig 2C showed reduced F-actin accumulation at the sealing zone (actin ring) under 30G (Fig 2F). These changes were more pronounced than observed under 3G, which are described in the following section. Additionally, nuclear positioning was strongly affected by hypergravity, with the proportion of nuclei on actin rings increasing from 34.15% at 1G to 83.56% at 30G, though the total number of nuclei per cell remained unchanged (Fig 2G, H). Osteoclast diameter showed no significant difference between 1G and 30G following short-term exposure (Fig 2I). These results indicate that short-term strong hypergravity induces rapid cytoskeletal remodeling and nuclear repositioning in osteoclasts.

### Effects of 3G hypergravity on osteoclast actin rings and bone resorption

**Fig 3:**
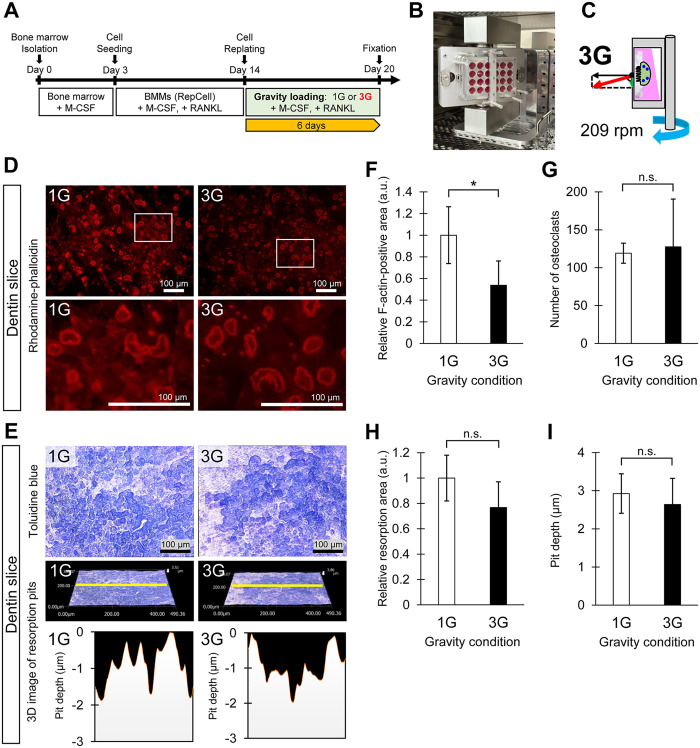
Moderate hypergravity (3G) reduces F-actin accumulation in osteoclasts on dentin. **(A)** Experimental timeline. BMM-derived osteoclasts differentiated on RepCell were transferred to dentin slices for pit assays. **(B)** CL-5100 hypergravity device with mounted 24-well plate. **(C)** Schematic diagram of gravity-generated mechanical loading under hypergravity applied to osteoclasts. **(D)** Representative osteoclasts on dentin slices stained with Rhodamine-phalloidin. Magnified views of the boxed regions are shown. **(E)** Representative resorption pits stained with Toluidine Blue, followed by 3D reconstruction. Yellow lines indicate cross-section positions. **(F)** Quantification of F-actin-positive area. ImageJ threshold values were set to 40-60, and cells with a diameter ≥20 µm were analyzed. **(G)** Osteoclast number was determined by manual counting of actin rings. **(H)** Quantification of pit area. Threshold values were set to 70-160, and pits with a diameter ≥10 µm and an aspect ratio <1.5 were analyzed. **(I)** Quantification of pit depth. Scale bar: 100 µm. Error bars indicate standard deviation (SD). Two-tailed unpaired t-test. *p < 0.05. Data were obtained from two independent experiments. Four dentin slices were analyzed per condition (n = 4 dentin slices). For image-based analyses, five fields per well were analyzed for panels F and G, and four fields per well for panels H and I.

Following the initial assessment at 30G, the effects of mild hypergravity (3G) were examined to evaluate potential impacts that may be more relevant to actual incidents of human exposure. To exclude any potential effects of hypergravity on the differentiation process, RepCell dishes were used to obtain premature osteoclasts that had already undergone differentiation prior to gravity loading. RepCell is a temperature-responsive culture system that allows intact osteoclasts to be detached without enzymatic treatment, ensuring preservation of cytoskeletal structures. BMM-derived osteoclasts were cultured on RepCell dishes, then transferred to dentin slices for pit assays at 1G or 3G for 6 days (Fig 3A) using a CL-5100 at 209 rpm (Fig 3B, C). When actin rings were exposed to 3G, the area of the sealing zone with fluorescence intensity above the predefined threshold was significantly reduced (Fig 3D, F), indicating that F-actin accumulation was decreased under hypergravity. In contrast, the total number of osteoclasts remained unchanged (Fig 3G). In resorption assays, neither the relative resorption area nor pit depth showed significant differences between the 1G and 3G groups (Fig 3E, H, I). In contrast, in osteoclast-osteoblast cocultures, pit depth was significantly reduced under 3G compared with 1G (S2 Fig.). These results suggest that modest 3G hypergravity induces significant alterations in sealing zone (actin ring) organization without affecting osteoclast number, and that attenuation of bone resorption, particularly in pit depth, becomes evident under osteoclast-osteoblast coculture conditions. To further distinguish the effects of hypergravity from those of force direction relative to the substrate, additional pit assays were performed under inverted loading conditions (1G, −1G, and −3G). Under these conditions, neither F-actin-positive area nor resorption pit depth differed significantly among the three groups (S3 Fig.), indicating that reversal of the gravity-generated force vector relative to the substrate did not impair osteoclast attachment or bone resorption on dentin slices.

### Short-term 3G hypergravity alters F-actin and nuclei distribution

**Fig 4:**
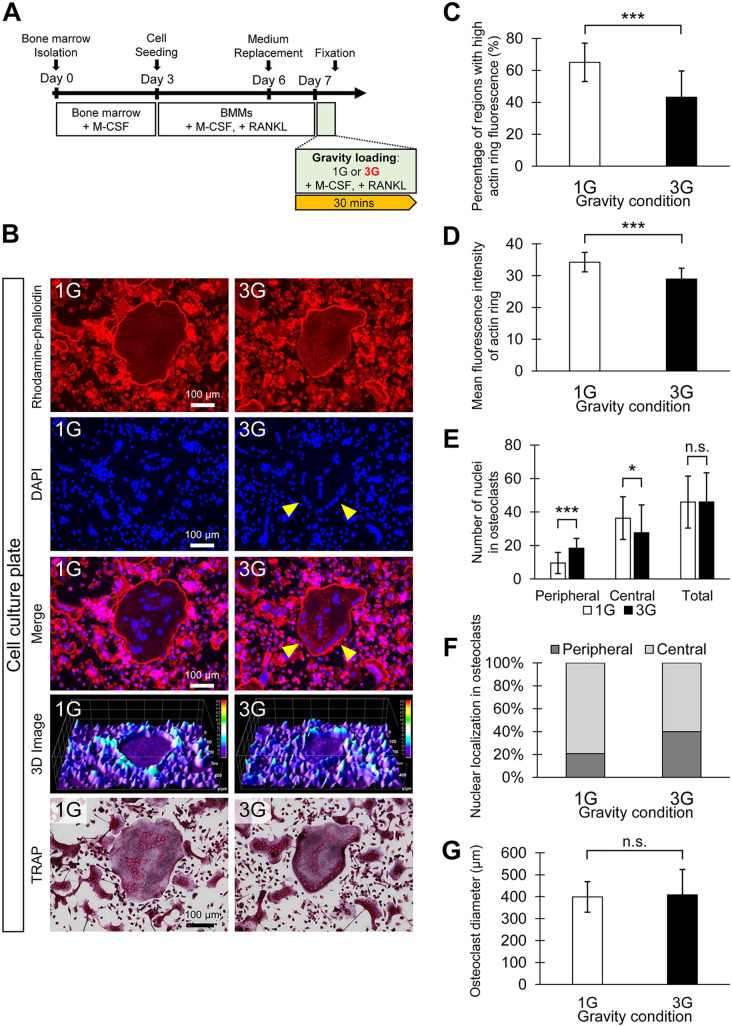
Mild effects of short-term (30 minutes) moderate hypergravity (3G) on F-actin and nuclei distribution. **(A)** Experimental timeline. Osteoclasts were exposed to 1G or 3G for 30 minutes. **(B)** Rhodamine-phalloidin and DAPI staining shown with merged images, 3D fluorescence intensity maps, and TRAP staining. Yellow arrows indicate nuclei located within actin rings. **(C)** Percentage of regions with high actin ring fluorescence. **(D)** Mean fluorescence intensity of the actin ring. **(E, F)** Representative images showing nuclear positioning **(E)** and ratio of peripheral vs central localization **(F)**. **(G)** Osteoclast diameter. Scale bar: 100 µm. Error bars indicate standard deviation (SD). Two-tailed unpaired t-test. *p < 0.05, ***p < 0.001. Data were obtained from two independent experiments. Panel D was analyzed using five wells, with three osteoclasts analyzed per well (n = 15 cells). Panels C and E-G were analyzed using eight wells, with three osteoclasts analyzed per well (n = 24 cells).

To examine the immediate effects of mild hypergravity, osteoclasts were exposed to 3G for 30 minutes (Fig 4A). Decreased fluorescence intensity of actin rings was shown in localized regions by Rhodamine-phalloidin staining (Fig 4B), and quantitative analyses demonstrated a reduction in both the proportion of regions with high actin ring fluorescence and the mean fluorescence intensity of the actin ring under 3G conditions (Fig 4C, D). Additionally, nuclear positioning was altered, as the proportion of nuclei localized on the sealing zone increased to 39.96% at 3G compared with 20.78% at 1G. However, the number of nuclei per cell and osteoclast diameter remained unchanged (Fig 4E-G). These findings suggest that even a low level of hypergravity rapidly affects cytoskeletal dynamics. To examine whether similar rapid cytoskeletal responses were observed at an intermediate gravity level, osteoclasts were exposed to 5G for 30 minutes. Consistent with the 3G condition, Rhodamine-phalloidin staining revealed a significant reduction in actin ring fluorescence under 5G (S4 Fig.). Quantitative analyses confirmed a decrease in mean actin ring fluorescence intensity. In addition, nuclear positioning was similarly affected, with an increased proportion of nuclei localized on the sealing zone under 5G (35.15%) compared with 1G (22.08%) (S4 Fig.). To further characterize cytoskeletal responses after gravity loading, osteoclasts were exposed to 5G for 30 minutes followed by incubation at 1G for an additional 30 minutes. Under these conditions, no significant differences were detected in actin ring fluorescence intensity, nuclear distribution, or osteoclast morphology compared with cells analyzed immediately after 5G exposure (S5 Fig.). Together, these findings demonstrate that short-term hypergravity rapidly induces cytoskeletal remodeling and changes in nuclear positioning in osteoclasts, and that similar responses are observed across multiple gravity conditions examined in this study. To further examine the contribution of force direction, osteoclasts were subjected to short-term inverted loading conditions (1G, −1G, and −3G) for 30 minutes. Both inverted static culture (−1G) and inverted centrifugation culture (−3G) induced cytoskeletal changes comparable to those observed under conventional hypergravity, including a significant reduction in the proportion of regions with high actin ring fluorescence and an increase in actin ring width (S6 Fig.). Mean actin ring fluorescence intensity was significantly reduced under −3G, whereas the decrease under −1G did not reach statistical significance. In addition, both inverted conditions caused pronounced peripheral redistribution of nuclei without altering the total number of nuclei per osteoclast or overall cell diameter (S6 Fig.). These findings indicate that osteoclast actin ring organization and nuclear positioning respond rapidly not only to substrate-directed compressive loading under conventional hypergravity, but also to tensile loading generated when the net force is directed away from the substrate under inverted loading conditions.

### Phosphoproteomic profiling of osteoclasts under hypergravity

**Fig 5:**
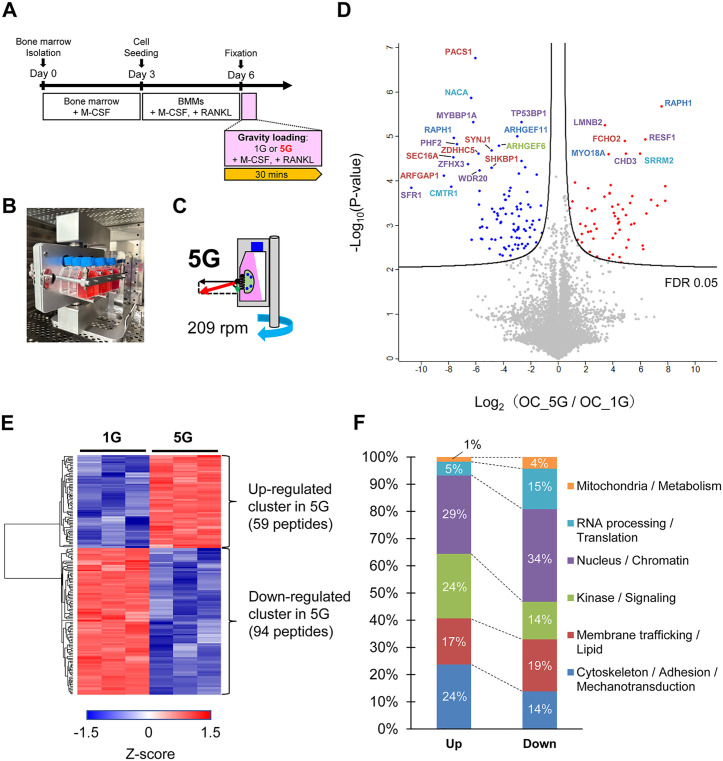
Phosphoproteomic profiling of osteoclasts exposed to a higher level of moderate hypergravity (5G) for 30 minutes. **(A)** Experimental timeline. **(B)** CL-5100 device with a mounted 25-mL flask. **(C)** Schematic diagram of gravity-generated mechanical loading under hypergravity applied to osteoclasts. **(D)** Volcano plot of phosphoproteomic analysis comparing 1G and 5G conditions (n = 3 per condition). Data were log2-transformed and imputed for missing values, followed by statistical testing using a two-sample t-test and permutation-based FDR correction (250 permutations; FDR = 0.05; S0 = 0.1). Red dots indicate significantly up-regulated phosphopeptides and blue dots indicate significantly down-regulated phosphopeptides (FDR < 0.05); gray dots indicate no significant change. Phosphopeptides with FDR < 0.01 are annotated by name. **(E)** Heatmap of differentially phosphorylated peptides identified in **(D)**. Phosphopeptides up-regulated under 5G conditions constitute the up-regulated cluster in 5G, whereas down-regulated phosphopeptides constitute the down-regulated cluster in 5G. Phosphopeptides with FDR < 0.05 were Z-score-normalized and visualized. **(F)** Bar graph summarizing the number of up-regulated (59 peptides) and down-regulated (94 peptides) phosphorylation events identified in **(D)**, with accompanying functional classification of the corresponding proteins.

To investigate early molecular events underlying hypergravity-induced sealing zone (actin ring) remodeling, osteoclasts were exposed to 5G hypergravity for 30 minutes using a CL-5100 centrifuge (Fig 5A-C). Phosphoproteomic analysis was performed with three independent biological replicates per condition (n = 3).

A total of 12,087 phosphopeptides were identified. Quantitative analysis revealed 59 phosphopeptides with significantly increased phosphorylation and 94 with decreased phosphorylation under hypergravity (Fig 5D; S1 Table). Differentially phosphorylated proteins were visualized by volcano plot analysis (Fig 5D) and hierarchical clustering heatmaps (Fig 5E), demonstrating clear separation between 1G and 5G conditions. Corresponding analyses based on unadjusted datasets are also shown (S7 Fig.).

Functional categorization of the differentially phosphorylated proteins showed that hypergravity predominantly affected six major cellular modules: (1) Cytoskeleton/ Cell adhesion/ Mechanotransduction, (2) Membrane trafficking/ Endocytosis/ Lipid signaling, (3) Kinase/ Phosphatase/ Signaling, (4) Nucleus/ Chromatin/ DNA repair, (5) RNA processing/ Translation, and (6) Mitochondria/ Metabolism/ Autophagy (Fig 5F). Among the upregulated phosphoproteins, substantial representation was observed in cytoskeleton/cell adhesion/mechanotransduction and kinase/phosphatase/signaling categories, together accounting for nearly half of the upregulated events, alongside a considerable fraction of nuclear and chromatin-associated proteins. This distribution suggests that hypergravity rapidly enhances phosphorylation events related to actin-membrane dynamics and intracellular signaling, while also engaging nuclear regulatory components. In contrast, downregulated phosphoproteins were more strongly biased toward nuclear/chromatin/DNA repair and RNA processing/translation categories, indicating preferential attenuation of phosphorylation within transcriptional and post-transcriptional regulatory pathways. Membrane trafficking-related proteins also constituted a notable fraction of downregulated phosphoproteins, suggesting functional rebalancing of vesicular and endocytic processes under hypergravity.

To assess the consistency between the exploratory and quantitative analyses, proteins commonly identified as differentially phosphorylated in both the dataset (n = 3) and the additional dataset (n = 1) were summarized (Table 1). An overview of the exploratory phosphoproteomic dataset is shown (S8 Fig.), and the complete list of identified phosphopeptides is provided (S2 Table). These overlapping proteins represent robust candidates for hypergravity-responsive phosphorylation events.

**Table 1:** Commonly regulated phosphorylation sites identified in both quantitative (n = 3) and exploratory (n = 1) phosphoproteomic analyses under hypergravity-induced mechanical loading conditions.

Up-regulated	Protein name	Protein symbol	Phosphorylation sites	Major cellular category	FC (5G/1G) (n = 3)	1G (n = 1)	5G (n = 1)
1	PI4P3-kinase C2 domain subunit alpha	PIK3C2A	620 S	Membrane trafficking/ Endocytosis/ Lipid signaling	14.13	0.00	5919.17
2	Rap guanine nucleotide exchange factor 2	RAPGEF2	1177 S	Kinase/ Phosphatase/ Signaling	3.27	0.00	8852.64
3	Tetratricopeptide repeat protein 39B	TTC39B	27 S	Membrane trafficking/ Endocytosis/ Lipid signaling	2.35	0.00	8293.27
**Down-regulated**	**Protein name**	**Protein symbol**	**Phosphorylation** **sites**	**Major cellular category**	**FC (5G/1G)** **(n = 3)**	**1G** **(n = 1)**	**5G** **(n = 1)**
1	DNA helicase	CHD3	765 S	Nucleus/ Chromatin/ DNA repair	0.04	40254.03	0.00
2	BRCA1-A complex subunit Abraxas 1	ABRAXAS1	48 S	Nucleus/ Chromatin/ DNA repair	0.05	10465.96	0.00
3	Ring finger protein 113A1	RNF113A1	251 S	Nucleus/ Chromatin/ DNA repair	0.07	19526.65	0.00
4	Ral guanine nucleotide dissociation stimulator-like 2	RGL2	762 S	Kinase/ Phosphatase/ Signaling	0.07	56830.08	0.00
5	Mitogen-activated protein kinase kinase kinase 1	MAP3K1	518 S	Kinase/ Phosphatase/ Signaling	0.10	15565.12	0.00
6	Rho guanine nucleotide exchange factor (GEF) 11	ARHGEF11	665 S	Cytoskeleton/ Cell adhesion/ Mechanotransduction	0.13	28994.73	0.00
7	T-complex protein 11-like protein 2	TCP11L2	31 S	RNA processing/ Translation	0.13	34847.02	0.00
8	Copine-1	CPNE1	54 S	Membrane trafficking/ Endocytosis/ Lipid signaling	0.13	6860.75	0.00
9	Centriolar coiled-coil protein of 110 kDa	CCP110	192 S	Nucleus/ Chromatin/ DNA repair	0.18	6248.29	0.00
10	E3 ubiquitin-protein ligase MSL2	MSL2	211 S	Nucleus/ Chromatin/ DNA repair	0.22	59674.61	0.00

This table summarizes phosphorylation sites that were commonly regulated in both the quantitative phosphoproteomic analysis (n = 3) and the exploratory phosphoproteomic analysis (n = 1) under hypergravity-induced mechanical loading conditions. The upper section lists commonly up-regulated phosphorylation sites, whereas the lower section lists commonly down-regulated phosphorylation sites identified in both datasets. The table includes protein name, protein symbol, phosphorylation sites, major cellular category, fold change (FC; 5G/1G) calculated from the quantitative dataset (n = 3), and quantitative phosphopeptide intensity values obtained from the exploratory analysis under 1G (n = 1) and 5G (n = 1) conditions. The intensity values for 1G (n = 1) and 5G (n = 1) represent phosphopeptide abundance measured by exploratory phosphoproteomic analysis without statistical filtering.

Together, these results demonstrate that short-term hypergravity induces a rapid and coordinated shift in phosphorylation states across cytoskeletal, membrane, and nuclear functional modules in osteoclasts.

## Discussion

### Hypergravity attenuates osteoclast bone-resorptive activity

The present study was conducted to comprehensively analyze the effects of hypergravity-induced mechanical loading on osteoclast bone-resorptive function and cytoskeletal organization, as well as the molecular basis underlying these responses, based on phosphoproteomic profiling. Important findings included the following: (i) prolonged exposure to 3G or 30G altered sealing zone (actin ring) structures and was associated with suppression of bone resorption; (ii) even short-term exposure (30 minutes) induced actin ring structural changes, resulting in decreased F-actin fluorescence, ring broadening, and nuclear peripheral repositioning; (iii) hypergravity induced coordinated changes in phosphorylation across multiple functional modules, including cytoskeletal organization, membrane trafficking, intracellular signaling, and nuclear/chromatin-associated regulatory proteins; and (iv) similar cytoskeletal and nuclear changes were also observed under inverted loading conditions (−1G and −3G), indicating that osteoclasts respond rapidly to changes in the magnitude and direction of gravity-generated mechanical forces.

The reduction in osteoclast resorptive activity caused by hypergravity is notable. While microgravity encountered during spaceflight has been shown to accelerate bone resorption and contribute to bone loss [27,28], the present findings suggest that increased gravity-generated mechanical loading may suppress osteoclast function. In particular, exposure to severe hypergravity at 30G resulted in significantly decreased resorption pit depth, indicating direct inhibition of osteoclast-mediated bone matrix degradation. Similarly, prolonged exposure to moderate hypergravity (3G) also reduced resorptive activity, demonstrating that this inhibitory effect is not restricted to extreme loading conditions. The present *in vitro* observations are consistent with previous *in vivo* findings showing that hypergravity suppressed bone resorption in ovariectomized rats [25]. Thus, these results suggest that osteoclasts show a bidirectional response to altered gravitational loading, with microgravity enhancing and hypergravity attenuating bone resorption, which highlights the role of mechanical cues in skeletal homeostasis. Although microgravity and hypergravity exert opposite effects on osteoclast activity, both conditions represent deviations from the normal gravitational environment and are likely to challenge cellular mechanosensing systems. In this regard, cytoskeletal organization may constitute a gravity-sensitive structural target that responds to altered mechanical loading, while downstream functional outcomes diverge depending on the direction and magnitude of the gravitational perturbation.

Importantly, however, in our experimental system, the functional consequences of altered gravitational loading were not uniform. Hypergravity significantly suppressed osteoclast bone-resorptive activity under upright loading conditions (3G and 30G), whereas resorptive activity appeared to be preserved under inverted loading conditions (−1G and −3G). These findings indicate that osteoclast function is not impaired simply by changes in gravitational orientation. Rather, suppression of bone resorption appears to depend on increased gravity-generated mechanical loading. Thus, while cytoskeletal remodeling may represent a common gravity-sensitive response, its downstream functional consequences are determined by the magnitude and direction of the mechanical forces generated by gravity.

### Cytoskeletal responses to altered gravity-generated forces

Structural changes occurring even after short-term hypergravity exposure indicate that osteoclasts have the capability to rapidly sense and respond to gravity-generated mechanical stress. It is likely that an altered F-actin assembly contributes to impaired adhesion and reduced resorptive activity under hypergravity-generated mechanical loading. The present pit assay results demonstrated that osteoclast migration was also suppressed by hypergravity conditions. The sealing zone (actin ring) has been shown to be closely associated with migration rate and resorption efficiency [29], thus their structural alterations observed in the present study may have a direct relationship with the reduced motility and functional capacity noted for osteoclasts.

Osteoclast activity is tightly regulated by osteoblast-derived cues, including RANKL/OPG signaling and contact-dependent interactions [30], which influence resorption efficiency as well as cytoskeletal organization. In the present study, suppression of resorption pit depth was observed in osteoclast-osteoblast cocultures under hypergravity conditions, suggesting that altered osteoblast-mediated signaling may contribute to the hypergravity-induced osteoclastic phenotype. At the same time, comparable alterations in actin ring organization were also detected in osteoclast monocultures, indicating that hypergravity directly affects osteoclasts in a cell-intrinsic manner, independent of osteoblast-derived regulation.

Another important finding is that nuclei were displaced onto actin rings under hypergravity. Such nuclear repositioning suggests modulation of intracellular tension, and potential involvement of the linker of nucleoskeleton and cytoskeleton (LINC) complex in cytoskeleton-nucleus coupling [31,32]. Similar findings have been reported for osteoblasts [33] and fibroblasts [34], but have rarely been described in osteoclasts.

Recent studies have begun to highlight the importance of intracellular spatial organization in multinucleated osteoclasts. In particular, proper clustering of centrosomes is required for polarized microtubule organization and efficient vesicular trafficking, which are essential for bone resorption [35]. Disruption of this organization leads to impaired cytoskeletal polarity and reduced resorptive activity. In addition, defective centrosome clustering has been shown to cause abnormal nuclear distribution and loss of polarized architecture in osteoclasts [36].

Actin filaments and podosome rings play critical roles in osteoclastogenesis and migration [37,38], and the present results further suggest that hypergravity-induced cytoskeletal remodeling may indirectly drive nuclear repositioning by altering intracellular force balance. Such aberrant nuclear localization could, in turn, interfere with actin-dependent processes by perturbing the spatial coordination required for sealing zone organization and polarized resorptive function.

Importantly, the inverted-loading experiments demonstrated that both inverted static culture (−1G) and inverted centrifugation (−3G) induced actin ring broadening and peripheral nuclear redistribution similar to those observed under conventional hypergravity. Osteoclasts remained firmly adherent to dentin slices under these inverted conditions and maintained bone-resorptive activity, with no significant differences in resorption pit depth among 1G, −1G, and −3G conditions. These findings further indicate that the observed cytoskeletal and nuclear responses are not primarily driven by substrate-directed compressive loading or hydrostatic pressure. If simple compression or medium-derived hydrostatic forces were the dominant determinants, opposite cellular responses would be expected under upright versus inverted orientations. Instead, the comparable phenotypes observed under both conditions support a model in which gravity-generated mechanical forces, despite opposite loading directions relative to the substrate, produce similar intracellular mechanical stresses and structural responses. Such effects may arise from differential displacement of intracellular components, including nuclei and organelles, thereby perturbing intracellular force balance throughout the multinucleated osteoclast. Although hydrostatic pressure may contribute as a secondary, uniformly distributed factor, it is unlikely to represent the principal driver of the observed structural changes.

Together, these findings point to a previously underappreciated aspect of osteoclast mechanotransduction, in which hypergravity perturbs both cytoskeletal organization and nucleo-cytoskeletal interactions, potentially leading to functional impairment. Importantly, the concurrent occurrence of actin remodeling and nuclear repositioning suggests that these structural responses are mechanically coupled rather than independent events. Although causality cannot be established in the present study, these findings suggest that cytoskeletal-nuclear reorganization may contribute to the functional impairment of osteoclasts under hypergravity-generated mechanical loading.

### Molecular basis revealed by phosphoproteomics

In this study, phosphoproteomic analysis with biological replication (n = 3) revealed that hypergravity induces broad yet structured changes in phosphorylation across multiple cellular systems, rather than isolated modulation of individual proteins. Differentially phosphorylated proteins were distributed among defined functional modules, including cytoskeletal organization, membrane trafficking, intracellular signaling, nuclear/chromatin regulation, and RNA processing, indicating that gravity-generated mechanical stress elicits a system-level reorganization of mechanotransduction pathways in osteoclasts. This modular pattern is consistent with established models of mechanotransduction, in which mechanical cues are integrated across interconnected cellular compartments rather than conveyed through single linear signaling cascades [39,40].

Quantitative categorization demonstrated that upregulated phosphorylation events were particularly enriched in cytoskeleton/cell adhesion/mechanotransduction and kinase/phosphatase/signaling modules, together accounting for a substantial fraction of the hypergravity-induced phosphorylation response. In parallel, a notable proportion of upregulated phosphoproteins was also associated with nuclear and chromatin-related functions, suggesting that hypergravity rapidly engages both peripheral structural networks and nuclear regulatory components. The enrichment of cytoskeletal regulators, membrane-cytoskeleton linkers, actin-myosin-associated proteins, and vesicle trafficking factors supports the notion that hypergravity enhances phosphorylation-dependent reinforcement and remodeling of actin-membrane architectures. These molecular changes are consistent with the rapid sealing zone reorganization and altered osteoclast morphology observed at the cellular level in this study, as well as with established roles of cytoskeletal regulation in osteoclast function [41].

In contrast, downregulated phosphorylation events exhibited a stronger relative bias toward nuclear/chromatin/DNA repair and RNA processing/translation modules. The prominence of chromatin remodelers, histone-modifying enzymes, transcriptional regulators, and RNA processing factors among downregulated phosphoproteins suggests that hypergravity preferentially attenuates or reprograms phosphorylation-dependent nuclear regulatory pathways. Notably, membrane trafficking and endocytic components also constituted a considerable fraction of downregulated proteins, indicating that hypergravity does not simply activate peripheral pathways but induces a coordinated rebalancing of phosphorylation across multiple cellular systems.

The concurrent representation of nuclear and chromatin-associated proteins in both up- and downregulated datasets suggests that hypergravity does not uniformly suppress nuclear signaling. Instead, it likely induces selective phosphorylation remodeling within nuclear regulatory networks. The presence of proteins involved in nucleo-cytoskeletal coupling, centrosome organization, and chromatin architecture among the differentially phosphorylated proteins further supports a model in which hypergravity alters force transmission along the membrane-cytoskeleton-nucleus axis. Such modulation may influence nuclear positioning, chromatin accessibility, and transcriptional responsiveness through phosphorylation-dependent mechanisms, in line with emerging concepts of nuclear mechanotransduction [39,40,42].

Taken together, these phosphoproteomic data indicate that hypergravity signaling in osteoclasts is characterized not by a simple dichotomy between cytoskeletal activation and nuclear suppression, but by a coordinated, module-specific reprogramming of phosphorylation states. Enhanced phosphorylation of cytoskeletal and signaling components, coupled with selective attenuation of nuclear and RNA regulatory phosphorylation, provides a molecular framework linking mechanical stress to structural adaptation and altered cellular function. This coordinated response may underlie the impaired osteoclast activity observed under hypergravity-generated mechanical loading [39–41].

Notably, a limited subset of differentially phosphorylated proteins was consistently detected in both the exploratory phosphoproteomic analysis (n = 1) and the quantitative analysis with biological replication (n = 3). Although the overlap was modest, these shared candidates were distributed across cytoskeletal, membrane trafficking, and nuclear regulatory modules, providing additional support for the involvement of these functional systems in the hypergravity response [43]. The phosphoproteomic dataset generated in this study will serve as a valuable resource for future studies aimed at validating and functionally characterizing individual hypergravity-responsive phosphorylation‌‌ events.

### Limitations and future directions

The present experiments were conducted *in vitro* and the effects of hypergravity on *in vivo* bone remodeling remain to be determined. In addition, it should be noted that the 3G and 30G experiments were performed using different hypergravity devices, which precludes direct quantitative comparison between these two conditions. Accordingly, the present study does not attempt to compare the magnitude of osteoclast responses between 3G and 30G. Nevertheless, qualitatively similar cytoskeletal changes, characterized by reduced F-actin fluorescence intensity and peripheral nuclear repositioning, were consistently observed across multiple hypergravity conditions, including 3G, 5G, and 30G. The reproducibility of these features across different gravity levels and experimental platforms suggests that they represent a common osteoclast response to gravity-generated mechanical loading, rather than device-specific effects. Future studies using a unified experimental system will be required to enable rigorous quantitative comparison across gravity magnitudes. Moreover, it remains unclear whether phosphorylation changes are causal for functional suppression or represent secondary responses. While disorganization of actin rings was induced by 30 minutes of hypergravity, the present data indicate that returning cells to 1G for 30 minutes was insufficient to restore actin ring fluorescence intensity to baseline levels (S5 Fig.). This suggests that recovery of cytoskeletal organization, if it occurs, may require longer periods under normal gravity. Further studies will be necessary to examine the time dependency and mechanisms underlying such potential reversibility, including targeted perturbation of candidate phosphoproteins, as well as extension of the analysis to in *vivo* models. Astronauts are instructed to perform exercise to mitigate microgravity-induced bone loss [44], underscoring the importance of mechanosensitive pathways in skeletal health.

A key consideration in the present experimental design is that osteoclasts cultured on dentin slices are exposed to distinct combinations of gravity-generated mechanical forces depending on sample orientation. Under conventional hypergravity, cells on the upper surface primarily experience substrate-directed compressive loading together with increased hydrostatic pressure from the overlying medium. In contrast, under inverted conditions, forces are directed away from the substrate, thereby imposing tensile loading while reducing compressive effects. During centrifugation, both upright and inverted samples are also subject to rotationally associated inertial forces, including minor shear-related components and Coriolis forces. However, these effects are absent under static inverted 1G (−1G) conditions. The observation that actin ring remodeling and nuclear redistribution occurred under both centrifuged and static inverted conditions indicates that these structural responses are not dependent on compressive loading or centrifugation-specific inertial forces alone. By contrast, the reduction in bone-resorptive activity observed only under conventional hypergravity suggests that compressive loading and/or increased hydrostatic pressure may be particularly important in suppressing osteoclast resorptive function. Future studies using systems that independently control compression, tension, hydrostatic pressure, and shear will be necessary to define the specific contributions of these individual mechanical stimuli.

## Conclusions

The present findings provide details showing attenuation of osteoclast bone-resorptive activity and altered cytoskeletal organization caused by hypergravity, as well as broad changes in phosphorylation signaling underlying those effects. These findings advance our understanding of how gravity-generated mechanical forces regulate osteoclast function and bone metabolism, and may contribute to the development of novel therapeutic strategies for spaceflight-induced bone loss as well as age-related osteoporosis.

## Supporting information

S1 Fig Microscopic images of osteoclasts immediately before and after hypergravity loading. (A) Experimental timeline. (B) Centrifuge used for hypergravity loading. (C) Schematic diagram indicating the direction of gravity-generated mechanical loading. (D) Representative microscopic image of osteoclasts before gravity loading. (E) Representative microscopic image of osteoclasts immediately after gravity loading. Red arrows indicate altered actin ring structures.
(TIF)

S2 Fig Moderate hypergravity (3G) in coculture attenuates pit depth of osteoclast-mediated bone resorption on dentin. (A) Experimental timeline. (B) Representative osteoclasts and osteoblasts cocultured on dentin slices stained with Rhodamine-phalloidin. Magnified views of the boxed regions are shown. (C) Representative resorption pits stained with Toluidine Blue, followed by 3D reconstruction. Yellow lines indicate cross-section positions. (D) Pit depth (µm). Scale bar: 100 µm. Error bars indicate standard deviation (SD). Two-tailed unpaired t-test. *p < 0.05. Data were obtained from two independent experiments. Four dentin slices were analyzed per condition (n = 4 dentin slices). For each dentin slice, quantitative analyses were performed using three randomly acquired images, and the averaged value per dentin slice was used for statistical analysis.
(TIF)

S3 Fig Inverted loading orientation does not impair osteoclast-mediated bone resorption on dentin. (A) Experimental timeline. (B) Schematic illustration of the gravity-generated mechanical loading conditions: conventional upright culture (1G), inverted static culture (−1G), and inverted centrifugation culture (−3G). (C) Representative images of osteoclasts cultured on dentin slices under 1G, −1G, and −3G conditions, stained with Rhodamine-phalloidin. (D) Representative resorption pits stained with Toluidine Blue. (E) Relative F-actin-positive area, normalized to the 1G control. (F) Pit depth (µm). Scale bar: 100 µm. Error bars indicate standard deviation (SD). One-way ANOVA followed by Tukey’s multiple comparison test. Data were obtained from two independent experiments. Eight dentin slices were analyzed per condition (n = 8 dentin slices). For each dentin slice, quantitative analyses were performed using three randomly acquired images, and the averaged value per dentin slice was used for statistical analysis.
(TIF)

S4 Fig Short-term exposure to moderate hypergravity (5G) induces changes in F-actin organization similar to those observed under 3G and 30G conditions. (A) Experimental timeline. Osteoclasts were exposed to 1G or 5G for 30 minutes. (B) Rhodamine-phalloidin and DAPI staining, shown with merged images. Yellow arrows indicate regions of partially decreased actin ring fluorescence. (C) Mean fluorescence intensity of actin rings. (D, E) Representative images showing nuclear positioning (D) and quantification of peripheral vs. central nuclear localization (E). Scale bar: 100 µm. Error bars indicate standard deviation (SD). Two-tailed unpaired t-test; *p < 0.05, **p < 0.01, ***p < 0.001. Two independent experiments were performed. One flask per condition was used in each experiment (two flasks in total), and a total of 27 cells were quantified (n = 27 cells).
(TIF)

S5 Fig Reversibility of hypergravity-induced changes in F-actin organization in osteoclasts. (A) Rhodamine-phalloidin and DAPI staining, shown with merged images. Yellow arrows indicate regions of partially decreased actin ring fluorescence. Scale bar: 100 µm. (B) Mean fluorescence intensity of actin rings. Error bars indicate standard deviation (SD). Two-tailed unpaired t-test. One independent experiment was performed. One flask per condition was used, and a total of 30 cells were quantified (n = 30 cells).
(TIF)

S6 Fig Short-term exposure to inverted loading orientation induces changes in F-actin organization and nuclear positioning in osteoclasts. (A) Experimental timeline. (B) Schematic illustration of the gravity-generated mechanical loading conditions: conventional upright culture (1G), inverted static culture (−1G), and inverted centrifugation culture (−3G). (C) Representative images of osteoclasts cultured on tissue culture plastic under 1G, −1G, and −3G conditions, stained with Rhodamine-phalloidin and DAPI, shown with merged images, three-dimensional reconstructions, and TRAP staining. (D) Percentage of regions with high actin ring fluorescence. (E) Actin ring area, normalized to the 1G control. (F) Mean fluorescence intensity of actin rings. (G) Number of nuclei per osteoclast. (H) Quantification of peripheral versus central nuclear localization. (I) Osteoclast diameter (µm). Scale bar: 100 µm. Error bars indicate standard deviation (SD). One-way ANOVA followed by Tukey’s multiple comparison test; *p < 0.05, **p < 0.01, ***p < 0.001. Data were obtained from two independent experiments. A total of 16 wells were analyzed across the two experiments, and three osteoclasts were quantified per well (n = 48 cells).
(TIF)

S7 Fig Overview of phosphoproteome changes in osteoclasts exposed to hypergravity (unadjusted data). (A) Volcano plot of phosphorylation levels. Phosphorylation data were obtained from a single experiment using three independent flasks per condition (1G and 5G). Peptides with fold change ≥2 and p < 0.05 are highlighted (red: upregulated, blue: downregulated, gray: unchanged). Y-axis: -Log10(p-value), X-axis: Log2(fold change). (B) Heatmap of Z-scored phosphorylation levels for peptides meeting the same criteria. Statistical analysis: t-test.
(TIF)

S8 Fig Exploratory phosphoproteomic analysis identifies up- and down-regulated phosphorylation sites (n = 1). (A) Experimental timeline. (B) CL-5100 device with a mounted 25-mL flask. (C) Schematic diagram of gravity-generated mechanical loading applied to osteoclasts. (D) Up- and down-regulated phosphorylation sites in the exploratory phosphoproteomic dataset (top 15 upregulated and top 15 downregulated phosphopeptides). FC: fold change.
(TIF)

S1 Table Differentially phosphorylated peptides identified by quantitative phosphoproteomic analysis (n = 3). This table lists phosphopeptides showing significantly altered phosphorylation under hypergravity (5G) compared with 1G, as identified by quantitative phosphoproteomic analysis with three independent biological replicates (n = 3). Peptides with a false discovery rate (FDR) < 0.05 were included, comprising 59 upregulated and 94 downregulated phosphopeptides. Information includes protein name, protein symbol, phosphorylation sites, fold change (FC), representative function, and major cellular category. Representative function: indicates the representative molecular role of each protein based on UniProt annotation and literature. Major cellular category denotes higher-order cellular systems relevant to mechanotransduction, manually curated to reflect cytoskeletal, membrane, nuclear, and metabolic organization.
(XLSX)

S2 Table Comprehensive list of phosphopeptides identified by exploratory phosphoproteomic analysis (n = 1). This table contains all phosphopeptides identified in the exploratory phosphoproteomic analysis performed with a single biological sample (n = 1). The dataset includes peptide sequence information, phosphorylation sites, corresponding master protein and gene symbols, quantitative intensity values under 1G and 5G conditions, and fold change (5G/1G). No statistical filtering was applied to this dataset.
(XLSX)
