# Supplementary figures and images for: Hypergravity reduces F-actin accumulation in osteoclasts, with attenuated bone resorption
Source: PLoS One. 2026 Jun 16;21(6):e0351542. doi: 10.1371/journal.pone.0351542 (PMC13271441; doi:10.1371/journal.pone.0351542)

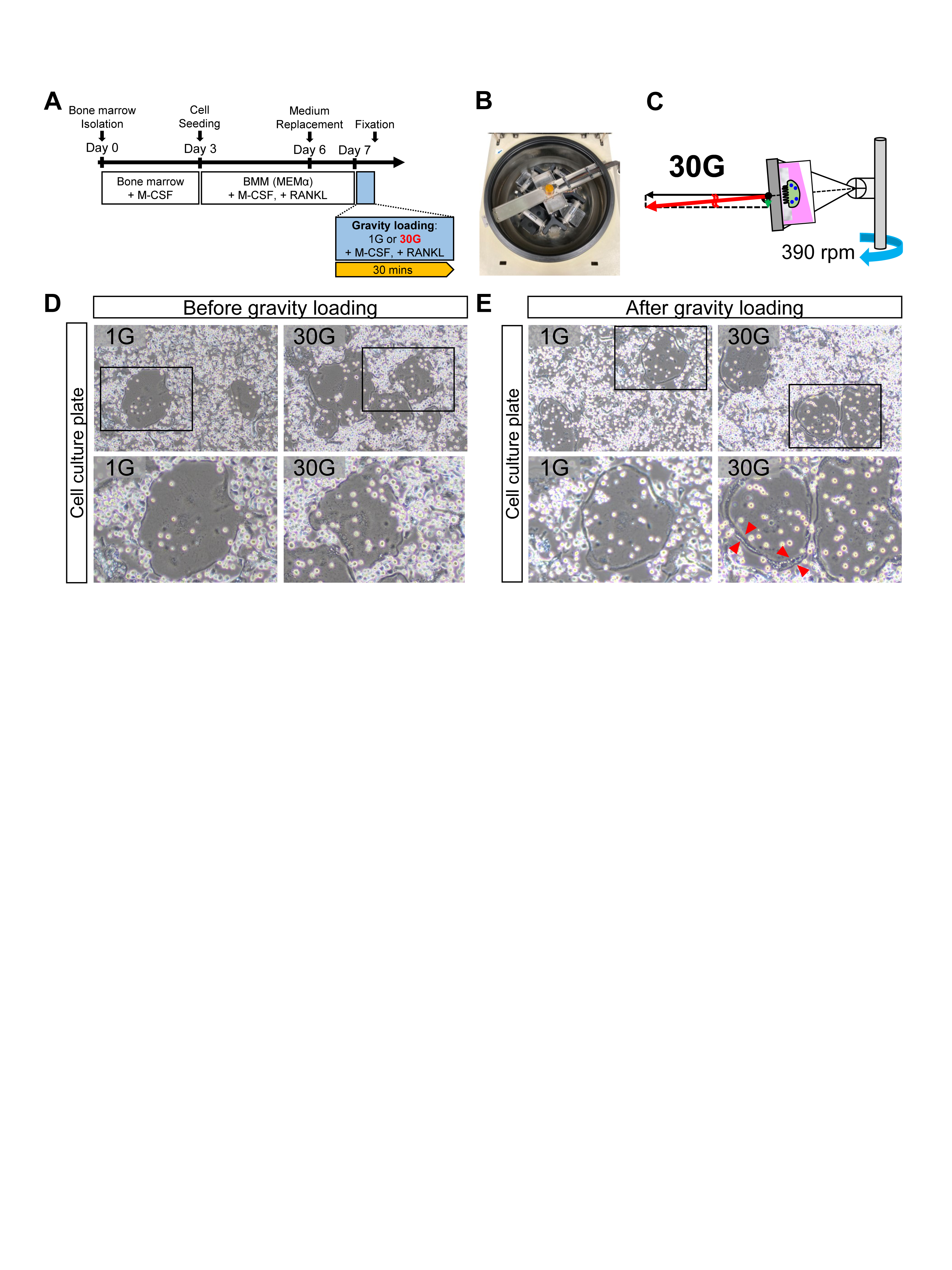

Supplement: S1 Fig — (A) Experimental timeline. (B) Centrifuge used for hypergravity loading. (C) Schematic diagram indicating the direction of gravity-generated mechanical loading. (D) Representative microscopic image of osteoclasts before gravity loading. (E) Representative microscopic image of osteoclasts immediately after gravity loading. Red arrows indicate altered actin ring structures. (TIF) [file pone.0351542.s001.tif]

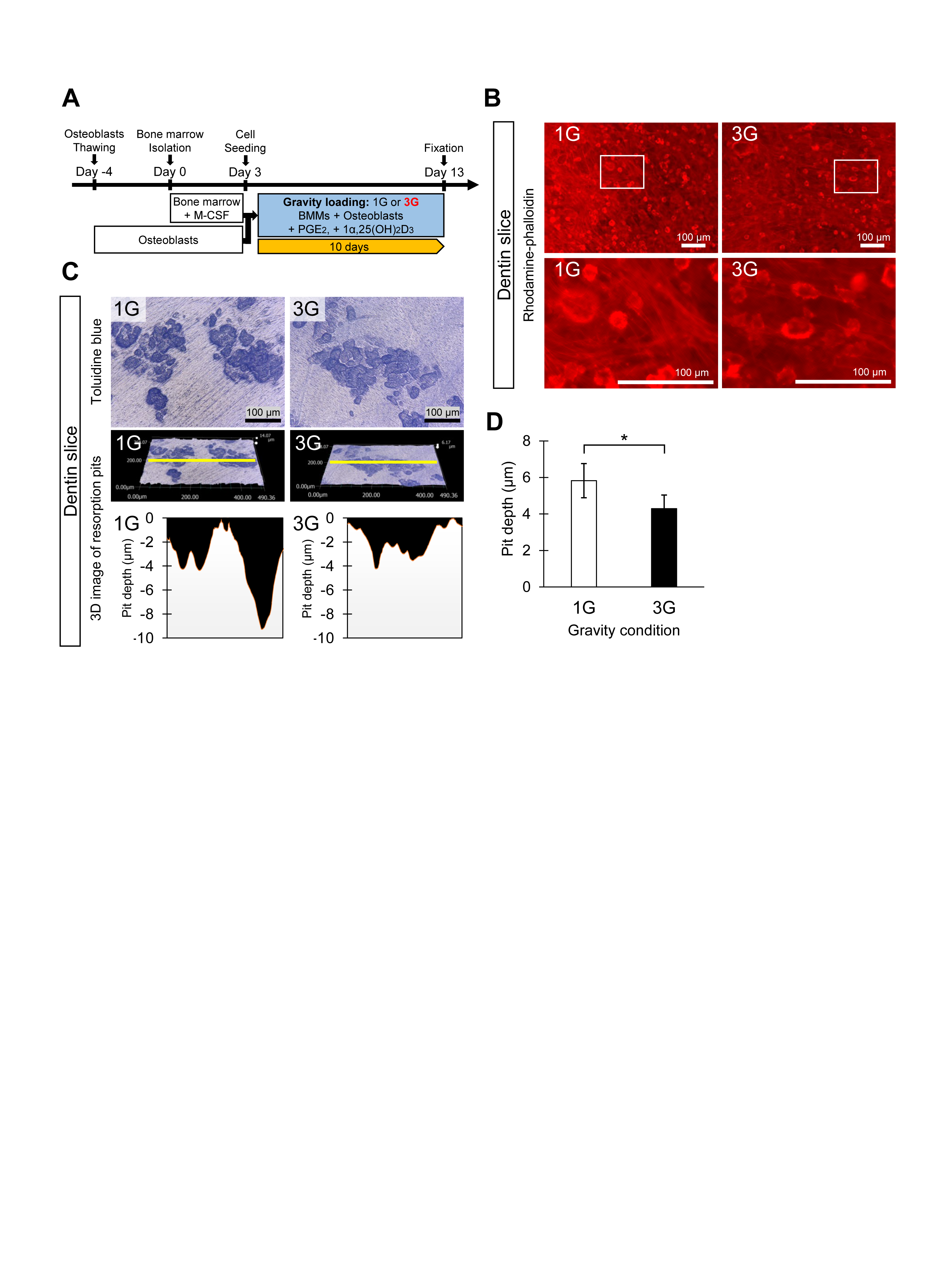

Supplement: S2 Fig — (A) Experimental timeline. (B) Representative osteoclasts and osteoblasts cocultured on dentin slices stained with Rhodamine-phalloidin. Magnified views of the boxed regions are shown. (C) Representative resorption pits stained with Toluidine Blue, followed by 3D reconstruction. Yellow lines indicate cross-section positions. (D) Pit depth (µm). Scale bar: 100 µm. Error bars indicate standard deviation (SD). Two-tailed unpaired t-test. *p < 0.05. Data were obtained from two independent experiments. Four dentin slices were analyzed per condition (n = 4 dentin slices). For each dentin slice, quantitative analyses were performed using three randomly acquired images, and the averaged value per dentin slice was used for statistical analysis. (TIF) [file pone.0351542.s002.tif]

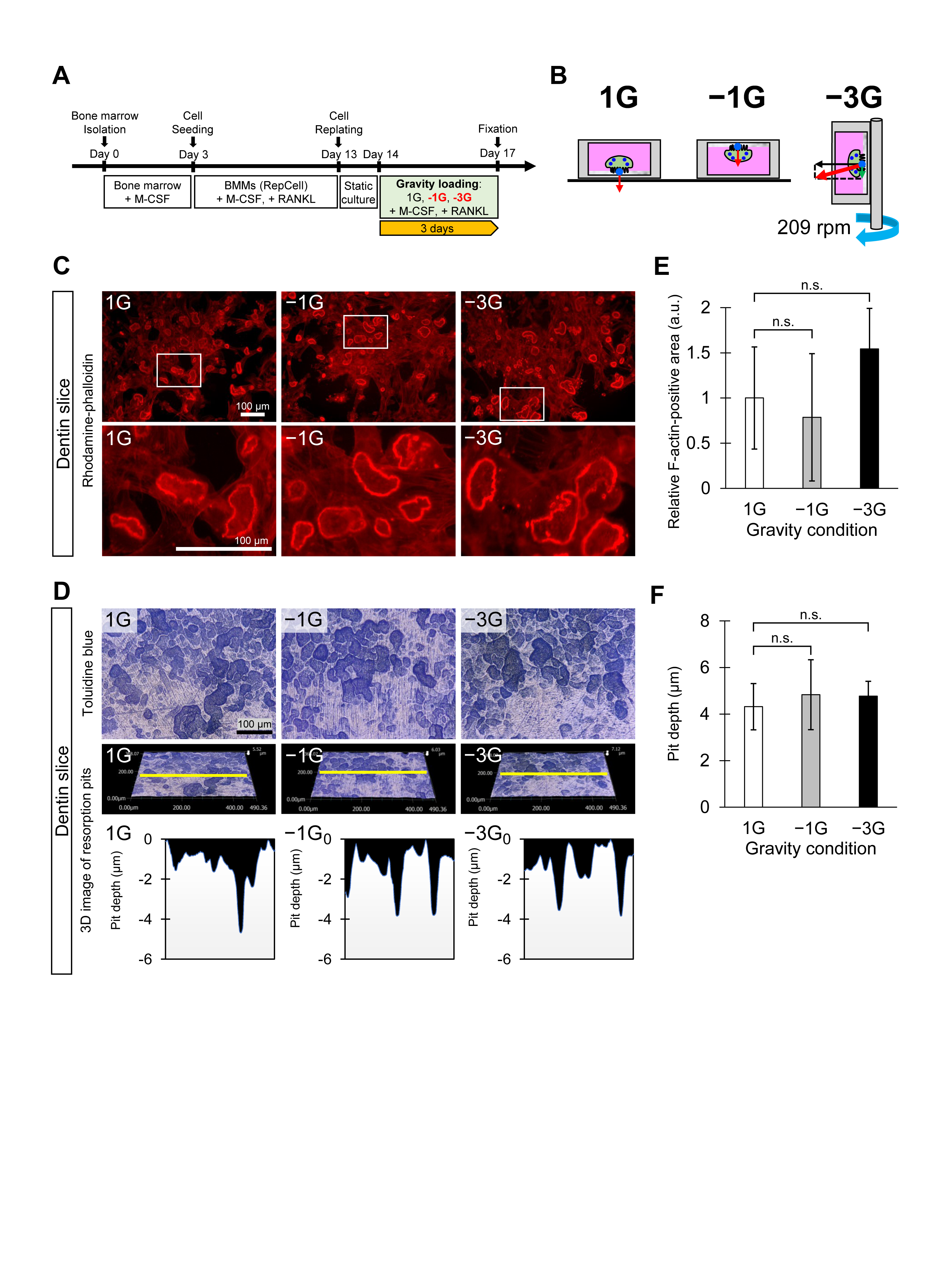

Supplement: S3 Fig — (A) Experimental timeline. (B) Schematic illustration of the gravity-generated mechanical loading conditions: conventional upright culture (1G), inverted static culture (−1G), and inverted centrifugation culture (−3G). (C) Representative images of osteoclasts cultured on dentin slices under 1G, −1G, and −3G conditions, stained with Rhodamine-phalloidin. (D) Representative resorption pits stained with Toluidine Blue. (E) Relative F-actin-positive area, normalized to the 1G control. (F) Pit depth (µm). Scale bar: 100 µm. Error bars indicate standard deviation (SD). One-way ANOVA followed by Tukey’s multiple comparison test. Data were obtained from two independent experiments. Eight dentin slices were analyzed per condition (n = 8 dentin slices). For each dentin slice, quantitative analyses were performed using three randomly acquired images, and the averaged value per dentin slice was used for statistical analysis. (TIF) [file pone.0351542.s003.tif]

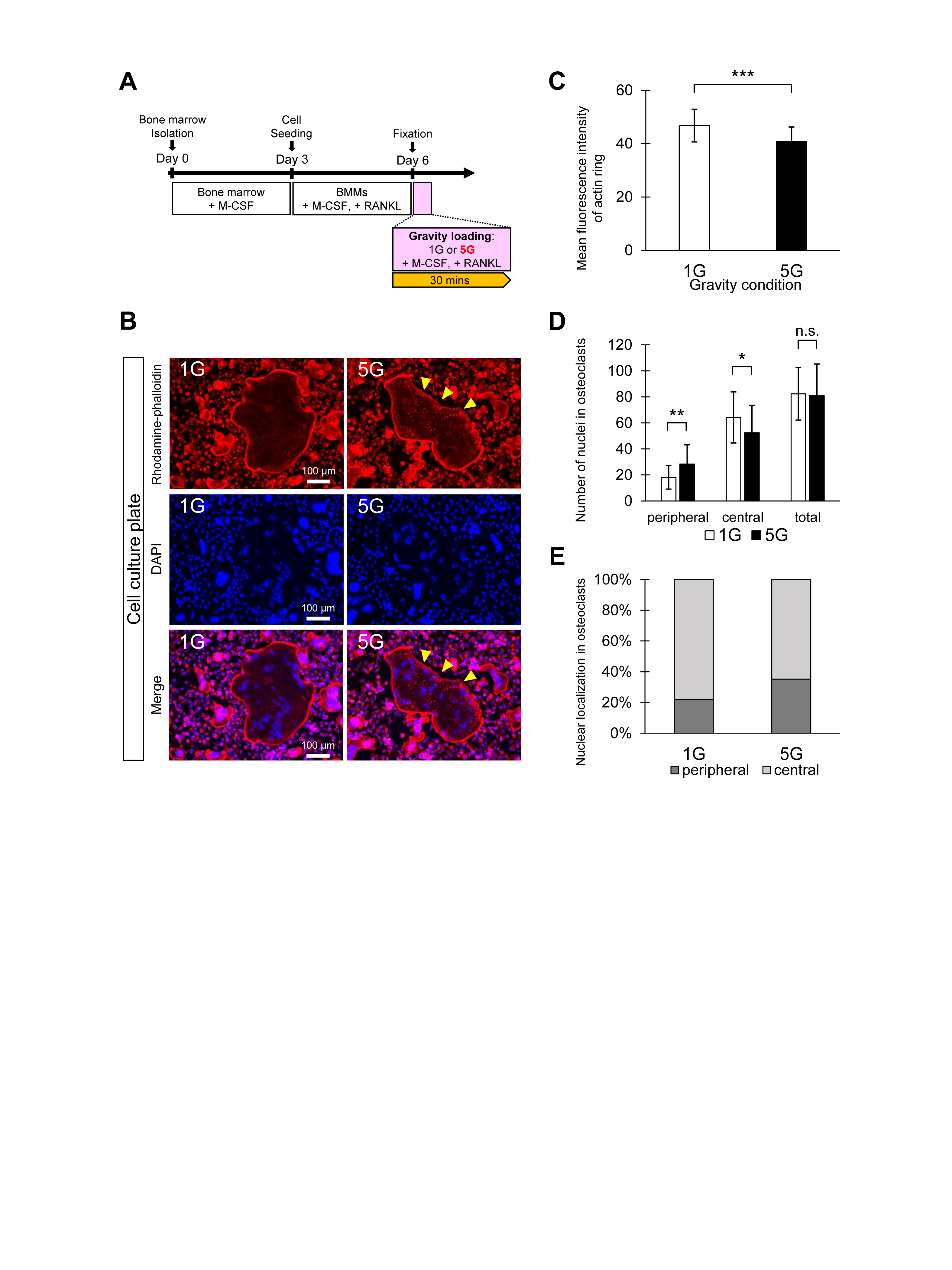

Supplement: S4 Fig — (A) Experimental timeline. Osteoclasts were exposed to 1G or 5G for 30 minutes. (B) Rhodamine-phalloidin and DAPI staining, shown with merged images. Yellow arrows indicate regions of partially decreased actin ring fluorescence. (C) Mean fluorescence intensity of actin rings. (D, E) Representative images showing nuclear positioning (D) and quantification of peripheral vs. central nuclear localization (E). Scale bar: 100 µm. Error bars indicate standard deviation (SD). Two-tailed unpaired t-test; *p < 0.05, **p < 0.01, ***p < 0.001. Two independent experiments were performed. One flask per condition was used in each experiment (two flasks in total), and a total of 27 cells were quantified (n = 27 cells). (TIF) [file pone.0351542.s004.tif]

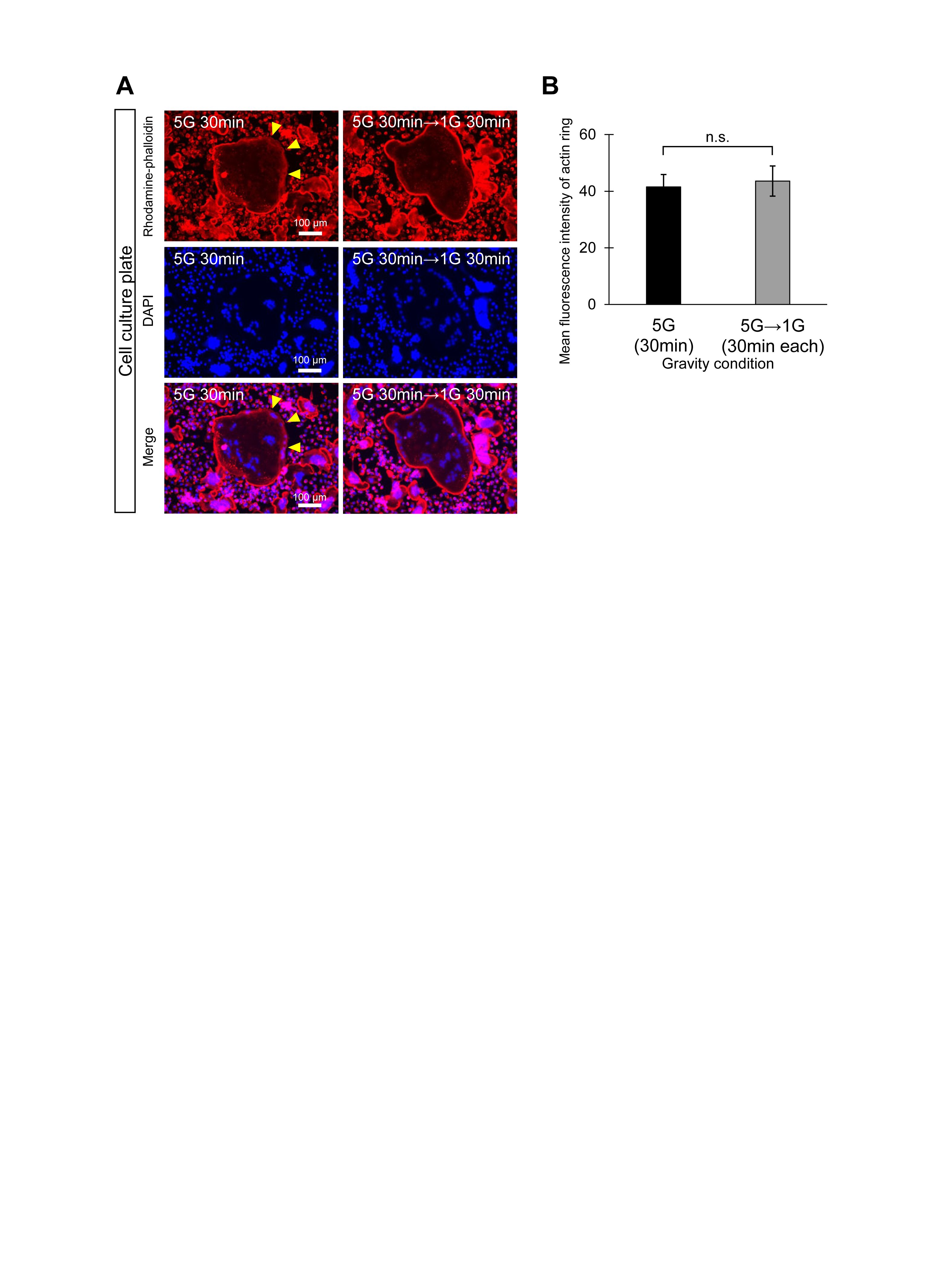

Supplement: S5 Fig — (A) Rhodamine-phalloidin and DAPI staining, shown with merged images. Yellow arrows indicate regions of partially decreased actin ring fluorescence. Scale bar: 100 µm. (B) Mean fluorescence intensity of actin rings. Error bars indicate standard deviation (SD). Two-tailed unpaired t-test. One independent experiment was performed. One flask per condition was used, and a total of 30 cells were quantified (n = 30 cells). (TIF) [file pone.0351542.s005.tif]

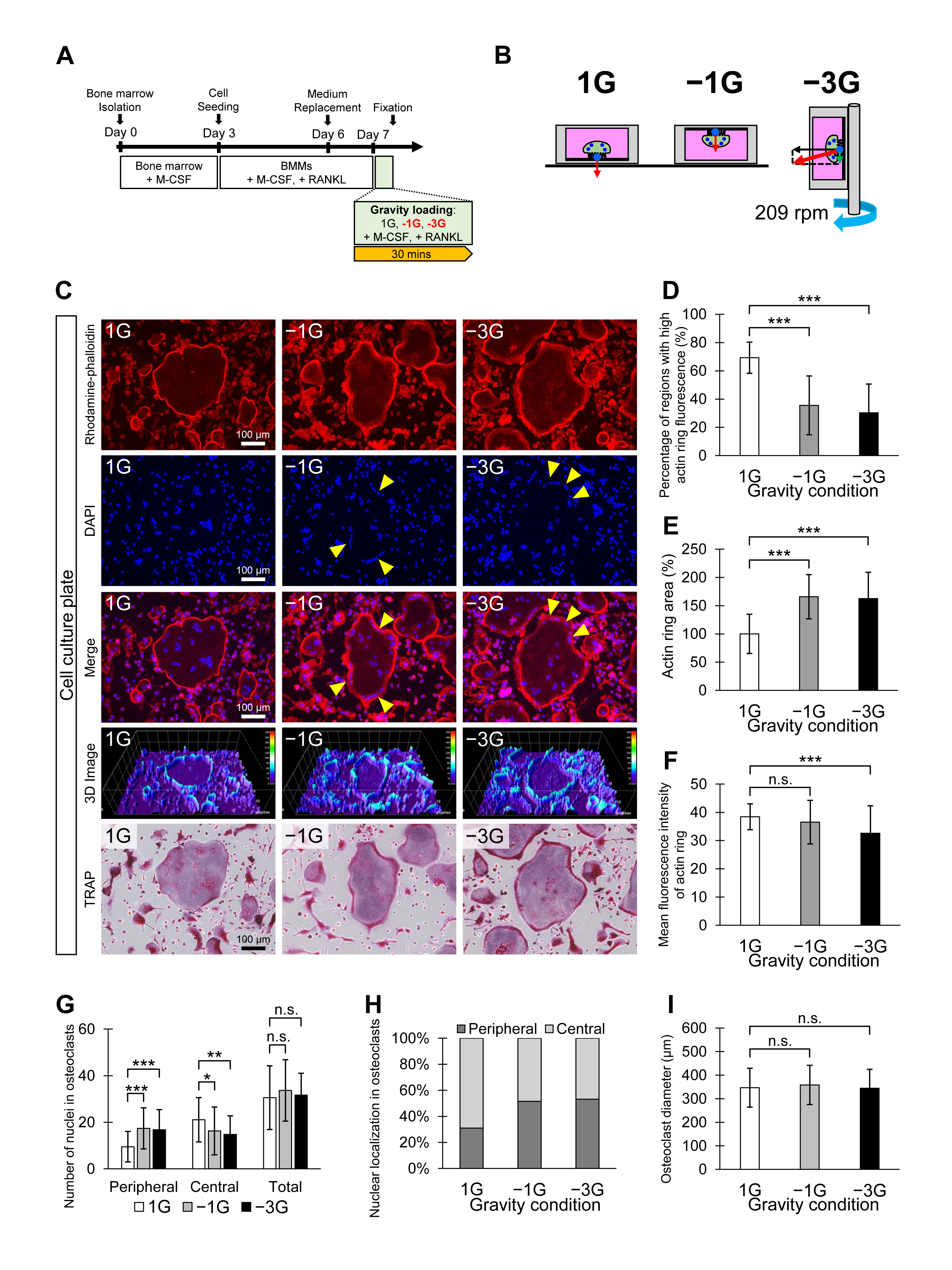

Supplement: S6 Fig — (A) Experimental timeline. (B) Schematic illustration of the gravity-generated mechanical loading conditions: conventional upright culture (1G), inverted static culture (−1G), and inverted centrifugation culture (−3G). (C) Representative images of osteoclasts cultured on tissue culture plastic under 1G, −1G, and −3G conditions, stained with Rhodamine-phalloidin and DAPI, shown with merged images, three-dimensional reconstructions, and TRAP staining. (D) Percentage of regions with high actin ring fluorescence. (E) Actin ring area, normalized to the 1G control. (F) Mean fluorescence intensity of actin rings. (G) Number of nuclei per osteoclast. (H) Quantification of peripheral versus central nuclear localization. (I) Osteoclast diameter (µm). Scale bar: 100 µm. Error bars indicate standard deviation (SD). One-way ANOVA followed by Tukey’s multiple comparison test; *p < 0.05, **p < 0.01, ***p < 0.001. Data were obtained from two independent experiments. A total of 16 wells were analyzed across the two experiments, and three osteoclasts were quantified per well (n = 48 cells). (TIF) [file pone.0351542.s006.tif]

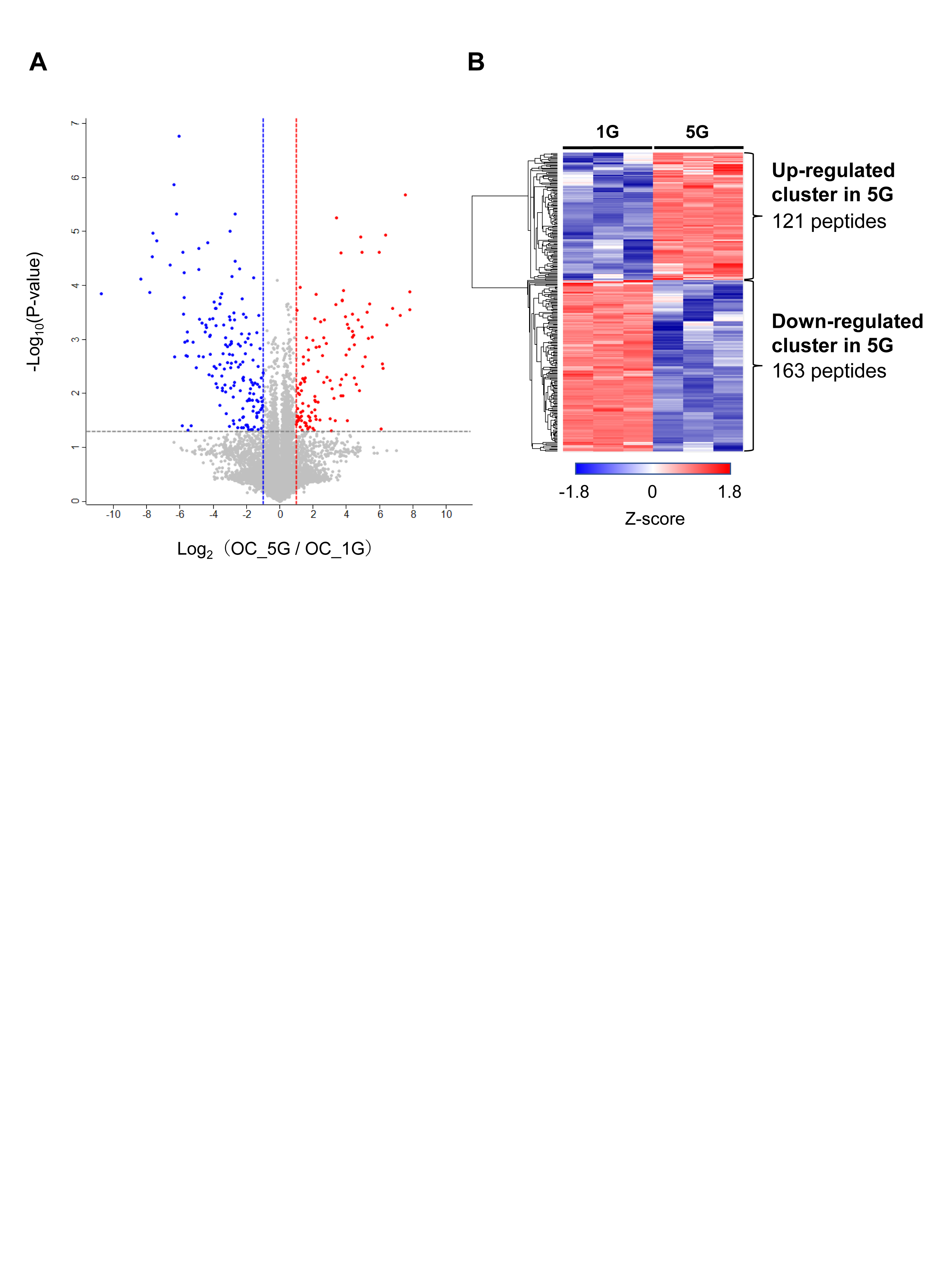

Supplement: S7 Fig — (A) Volcano plot of phosphorylation levels. Phosphorylation data were obtained from a single experiment using three independent flasks per condition (1G and 5G). Peptides with fold change ≥2 and p < 0.05 are highlighted (red: upregulated, blue: downregulated, gray: unchanged). Y-axis: -Log10(p-value), X-axis: Log2(fold change). (B) Heatmap of Z-scored phosphorylation levels for peptides meeting the same criteria. Statistical analysis: t-test. (TIF) [file pone.0351542.s007.tif]

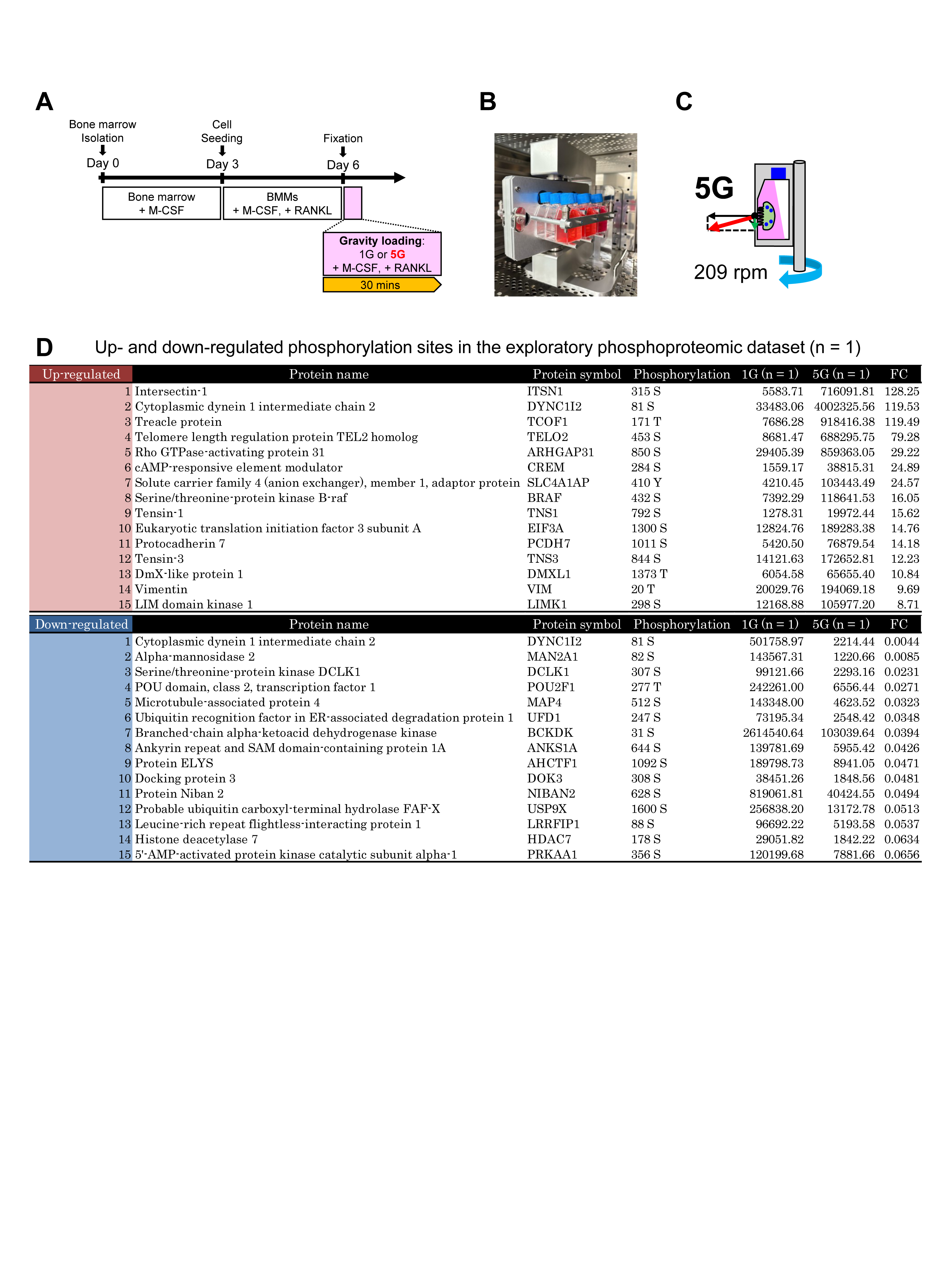

Supplement: S8 Fig — (A) Experimental timeline. (B) CL-5100 device with a mounted 25-mL flask. (C) Schematic diagram of gravity-generated mechanical loading applied to osteoclasts. (D) Up- and down-regulated phosphorylation sites in the exploratory phosphoproteomic dataset (top 15 upregulated and top 15 downregulated phosphopeptides). FC: fold change. (TIF) [file pone.0351542.s008.tif]
